# Pharmacological Inhibition of PIP4K2 Potentiates Venetoclax-Induced Apoptosis in Acute Myeloid Leukemia

**DOI:** 10.3390/ijms242316899

**Published:** 2023-11-29

**Authors:** Keli Lima, Maria Fernanda Lopes Carvalho, Diego Antonio Pereira-Martins, Frederico Lisboa Nogueira, Lívia Bassani Lins de Miranda, Mariane Cristina do Nascimento, Rita de Cássia Cavaglieri, Jan Jacob Schuringa, João Agostinho Machado-Neto, Eduardo Magalhães Rego

**Affiliations:** 1 Laboratory of Medical Investigation in Pathogenesis and Targeted Therapy in Onco-Immuno-Hematology (LIM-31), Department of Internal Medicine, Hematology Division, Faculdade de Medicina, University of São Paulo, São Paulo CEP 13566-590, Brazil; kelilima@usp.br (K.L.); d.a.pereira.martins@umcg.nl (D.A.P.-M.); fredericoln@gmail.com (F.L.N.); mariane.cristina.nascimento@usp.br (M.C.d.N.); 2 Department of Pharmacology, Institute of Biomedical Sciences, University of São Paulo, São Paulo CEP 13566-590, Brazil; mfernandalopes@usp.br (M.F.L.C.); liviamirands@usp.br (L.B.L.d.M.); r.cavaglieri@hc.fm.usp.br (R.d.C.C.); jamachadoneto@usp.br (J.A.M.-N.); 3 Department of Experimental Hematology, University of Groningen, 9718 BG Groningen, The Netherlands; j.j.schuringa@umcg.nl

**Keywords:** acute myeloid leukemia, phosphatidylinositol-5-phosphate 4-kinase type 2, THZ-P1-2, venetoclax

## Abstract

Phosphatidylinositol-5-phosphate 4-kinase type 2 (PIP4K2) protein family members (PIP4K2A, PIP4K2B, and PIP4K2C) participate in the generation of PIP*4*,*5*P_2_, which acts as a secondary messenger in signal transduction, a substrate for metabolic processes, and has structural functions. In patients with acute myeloid leukemia (AML), high *PIP4K2A* and *PIP4K2C* levels are independent markers of a worse prognosis. Recently, our research group reported that THZ-P1-2 (PIP4K2 pan-inhibitor) exhibits anti-leukemic activity by disrupting mitochondrial homeostasis and autophagy in AML models. In the present study, we characterized the expression of PIP4K2 in the myeloid compartment of hematopoietic cells, as well as in AML cell lines and clinical samples with different genetic abnormalities. In ex vivo assays, PIP4K2 expression levels were related to sensitivity and resistance to several antileukemia drugs and highlighted the association between high *PIP4K2A* levels and resistance to venetoclax. The combination of THZ-P1-2 and venetoclax showed potentiating effects in reducing viability and inducing apoptosis in AML cells. A combined treatment differentially modulated multiple genes, including *TAp73*, *BCL2*, *MCL1*, and *BCL2A1*. In summary, our study identified the correlation between the expression of PIP4K2 and the response to antineoplastic agents in ex vivo assays in AML and exposed vulnerabilities that may be exploited in combined therapies, which could result in better therapeutic responses.

## 1. Introduction

Acute myeloid leukemia (AML) is an aggressive hematological neoplasm characterized by the proliferation, survival, and accumulation of blasts in the bone marrow, peripheral blood, and other organs, which suppresses normal hematopoiesis [1,2]. Several factors influence the prognosis of AML, including clinical and laboratory characteristics, as well as the choice and response to treatment, among others [3]. In recent years, significant advances in the understanding of the cellular and molecular biology of AML have been made, paving avenues for the identification and approval of new drugs for the treatment of the disease [4,5].

Recently, our research group identified the fact that phosphatidylinositol-5-phosphate 4-kinase type 2 (PIP4K2) inhibitors could be a new class of drugs with antileukemic potential [6]. PIP4K2 (PIP4K2A, PIP4K2B, and PIP4K2C) are lipid kinases that phosphorylate phosphatidylinositol (PI)5 P at position four of the inositol ring and generate PI*4*,*5*P_2_, which acts as a secondary messenger in signal transduction, a substrate for metabolic processes, and has structural functions [7,8]. In experimental models of AML, PIP4K2A was identified as a key protein for the maintenance of leukemia-initiating cells, acting on proliferation, clonogenicity, and survival [9]. A high *PIP4K2A* or *PIP4K2C* negatively impacts survival outcomes in a univariate analysis, but the combination of the expression of PIP4K2A and PIP4K2C was an independent prognostic factor in patients with AML [10]. THZ-P1-2 is a pan-inhibitor of PIP4K2 and selectively reduces viability through the disruption of mitochondrial homeostasis and changes in the autophagic flux [6].

The objective of this study was to comprehensively characterize the expression profile of PIP4K2 in normal and malignant hematopoietic cells, including myeloid cell lines. Furthermore, our investigation aimed to elucidate the role of PIP4K2 in the response to antineoplastic drugs within AML models. Consequently, we endeavor to propose promising pharmacological combinations incorporating the PIP4K2 inhibitor, THZ-P1-2, for combined therapeutic strategies.

## 2. Results

### 2.1. Expression of PIP4K2 Genes in Normal and Leukemic Hematopoiesis

**Figure 1:**
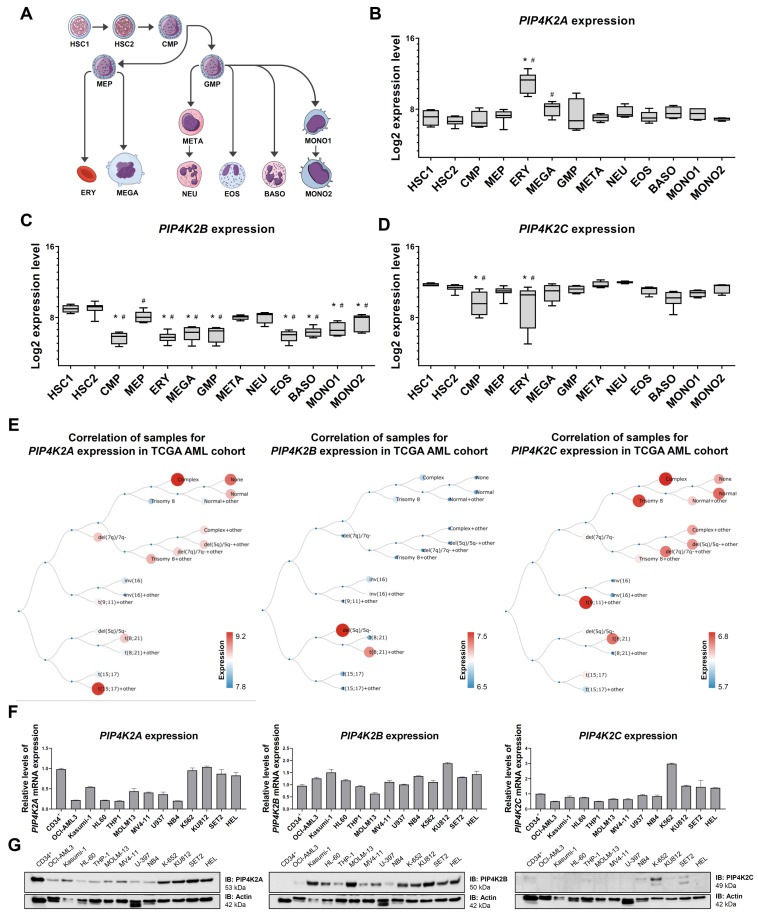
Expression of *PIP4K2A*, *PIP4K2B*, and *PIP4K2C* in normal and malignant hematopoietic cells. (**A**) Graphical legend for the myeloid differentiation hierarchy illustrating the analyzed cell subpopulations used in the analysis (https://mindthegraph.com/ (accessed on 2 August 2023)). Abbreviations: HSC, hematopoietic stem cells; CMP, common myeloid progenitor; GMP, granulocyte macrophage progenitor; MEP, megakaryocyte/erythrocyte progenitor; ERY, erythrocytes; MEGA, megakaryocytes; META, metamyelocytes; NEU, neutrophils; EOS, eosinophils; BASO, basophils; and MONO, monocytes. (**B**–**D**) Gene expression profile of *PIP4K2A* (probe 205570_at), *PIP4K2B* (probe 201080_at), and *PIP4K2C* (probe 218942_at) in myeloid cell subpopulations (GSE24759). The *p*-values and cell lineages are indicated in the graphs: * *p* < 0.05 cell lineage vs. HSC1, *^#^* *p* < 0.01 cell lineage vs. HSC2; ANOVA and Bonferroni post hoc test. (**E**) Schematic representation of *PIP4K2A*, *PIP4K2B*, and *PIP4K2C* expression in the different molecular subtypes of AML obtained from the BloodSpot Database (https://servers.binf.ku.dk/bloodspot/ (accessed on 2 August 2023)). (**F**) *PIP4K2A*, *PIP4K2B*, and *PIP4K2C* mRNA levels in normal CD34^+^ cells and AML cell lines. *ACTB* and *HPRT1* were used as the reference genes, and CD34^+^ cells were used as a calibrator. (**G**) Western blot analysis of the PIP4K2A, PIP4K2B, and PIP4K2C levels in the total cell extracts from CD34^+^ cells and AML cell lines; the membranes were re-probed with the antibody for the detection of actin and developed with the SuperSignal™ West Dura Extended Duration Substrate system using a G: BOX Chemi XX6 gel document system.

The transcript levels of *PIP4K2A*, *PIP4K2B*, and *PIP4K2C* were characterized in different myeloid cell subpopulations. *PIP4K2A* expression was higher in erythroid (ERY) and megakaryocytic (MEGA) cells compared to common myeloid progenitors (CMP), granulocyte-macrophage progenitors (GMP), megakaryocyte-erythrocyte progenitors (MEP), metamyelocytes (META), neutrophils (NEU), eosinophils (EOS), basophils (BAS), and monocytes (MONO). *PIP4K2B* expression was higher in hematopoietic stem cells (HSC). *PIP4K2C* expression was lower in CMP and ERY cells (Figure 1A–D). In patients with AML, *PIP4K2A* and *PIP4K2C* expression was higher in complex karyotype groups, whereas *PIP4K2B* expression was higher in del(5q)/5q- and t(8;21)+others groups (Figure 1E). In the myeloid leukemia cell lines, PIP4K2A expression was higher in K562, KU812, HEL, and SET2 cells, while the expression of PIP4K2B and PIP4K2C was similar among the evaluated cells, highlighting a high gene and protein expression of PIP4K2C in K562 cells (Figure 1F–G).

### 2.2. PIP4K2 mRNA Levels Are Associated with Drug Sensitivity in Silico Analysis from Ex Vivo Assays Data in Acute Myeloid Leukemia

**Figure 2:**
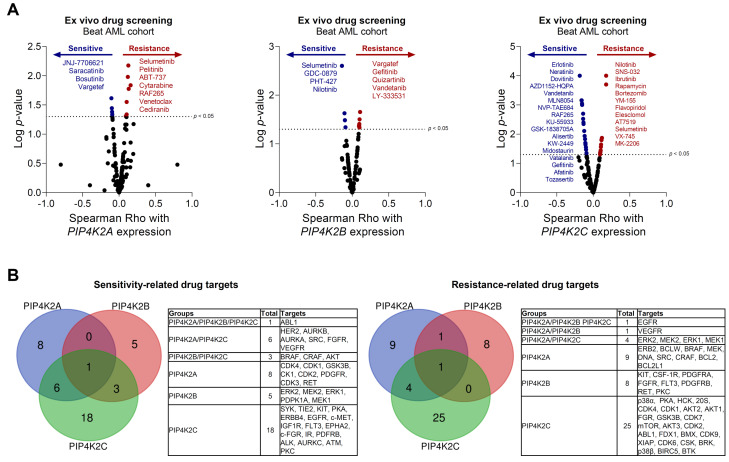
*PIP4K2A*, *PIP4K2B*, and *PIP4K2C* impact on drug sensitivity in ex vivo assays of acute myeloid leukemia cells. (**A**) Drug sensitivity according to *PIP4K2A*, *PIP4K2B*, and *PIP4K2C* expression in ex vivo assays of acute myeloid leukemia. Drugs with *p* < 0.05 are indicated using the Spearman correlation test. (**B**) The molecular targets (proteins/genes) of the identified drugs are listed and analyzed using Venn diagrams (http://bioinformatics.psb.ugent.be/ (accessed on 2 August 2023)).

Next, we evaluated the association of the PIP4K2 genes with the response to antineoplastic agents evaluated in the primary samples from AML patients. Using the Beat AML study [11,12] dataset, the transcriptional levels of *PIP4K2A* were positively correlated with the resistance to seven drugs (selumetinib, pelitinib, ABT-737, cytarabine, RAF265, venetoclax, and cediranib) and negatively correlated with four (JNJ-7706621, saracatinib, bosutinib, and vargetef). *PIP4K2B* expression was positively correlated with the resistance to five drugs (vargatef, gefitinib, quizartinib, vandetanib, and LY-333531) and negatively correlated with four (selumetinib, GDC-0879, PHT-427, and nilotinib). *PIP4K2C* expression was positively correlated with the resistance to twelve drugs (nilotinib, SNS-032, ibrutinib, rapamycin, bortezomib, YM-155, flavopiridol, elesclomol, AT7519, selumetinib, VX-745, and MK-2206) and negatively correlated with seventeen (erlotinib, neratinib, dovitinib, AZD1152-HQPA, vandetanib, MLN8054, NVP-TAE684, RAF265, KU-55933, GSK-1838705A, alisertib, KW-2449, midostaurin, vatalanib, gefitinib, afatinib, and tozasertib; Figure 2A). By analyzing the molecular targets of the identified drugs, it was identified that a high frequency of molecular targets is mutually exclusive between the *PIP4K2* genes associated with drug sensitivity and resistance. ABL1 was identified as a molecular target associated with sensitivity, related to the three genes, while EGFR was identified as a target associated with resistance, related to the *PIP4K2* genes (Figure 2B).

### 2.3. THZ-P1-2, a PIP4K2 Pan-Inhibitor, Augments Venetoclax-Induced Apoptosis

**Figure 3:**
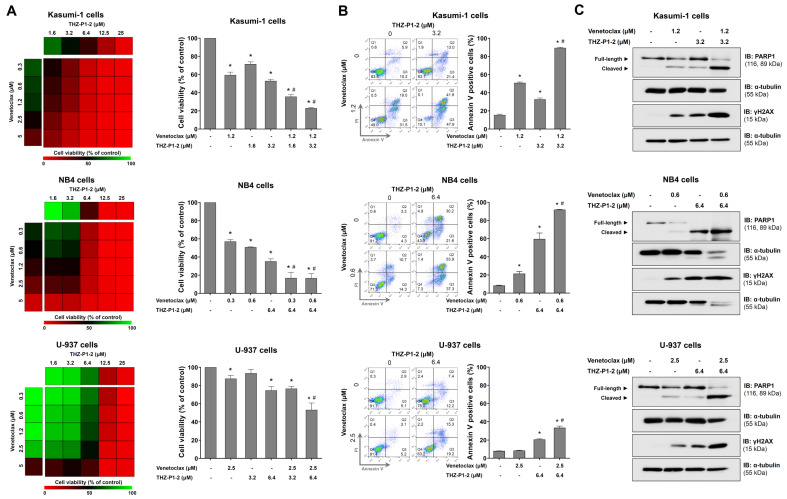
THZ-P1-2 potentiates venetoclax-induced apoptosis in acute myeloid leukemia cells. (**A**) Dose–response cytotoxicity for combined treatment was analyzed using a MTT assay for Kasumi-1, NB4, and U-937 cells treated with vehicle or graded concentrations of THZ-P1-2 (0.3, 0.6, 1.2, 2.5, and 5 µM) and venetoclax (1.6, 3.2, 6.4, 12.5, and 25 µM) alone or in combination with each other for 48 h, as indicated. The values are expressed as the percentage of viable cells for each condition relative to the vehicle-treated cells. The results are shown as the mean of at least three independent experiments. The bar graphs represent the cell viability for the selected concentrations. The *p*-values and cell lines are indicated as follows: * *p* < 0.05 treatment versus vehicle and ^#^ *p* < 0.05 monotherapy versus combined therapy; ANOVA test and Bonferroni post-test. (**B**) The apoptosis was detected using flow cytometry in Kasumi-1, NB4, and U-937 cells treated with THZ-P1-2 and/or venetoclax for 48 h using an APC-annexin V/PI staining method. Representative dot plots are shown for each condition; the upper and lower right quadrants (Q2 plus Q3) cumulatively contain the apoptotic population (annexin V+ cells). The bar graphs represent the mean ± SD of at least three independent experiments. The *p* values and cell lines are indicated in the graphs as follows: * *p* < 0.05 for THZ-P1-2- and/or venetoclax-treated cells vs. vehicle-treated cells and ^#^ *p* < 0.05 for THZ-P1-2- or venetoclax-treated cells versus the combination treatment at the corresponding doses; ANOVA and Bonferroni post-test. (**C**) Western blot analysis PARP1 (total and cleaved) and γH2AX levels in the total cell extracts from Kasumi-1, NB4, and U-937 cells treated with vehicle, THZ-P1-2, and/or venetoclax at the indicated concentrations; the membranes were re-probed with the antibody for the detection of α-tubulin and developed with the SuperSignal™ West Dura Extended Duration Substrate system (Thermo Fisher Scientific, Cleveland, OH, USA) using a G: BOX Chemi XX6 gel document system.

Among the drugs identified as being associated with resistance in the presence of high levels of *PIP4K2A* expression, venetoclax draws attention. Venetoclax is a selective BCL2 inhibitor that was recently introduced into AML therapy and has been successful in the treatment of patients who were ineligible for intensive chemotherapy [13]. THZ-P1-2 serves as a pan-inhibitor for PIP4K2 using covalently binding to a specific cysteine residue situated within a disordered loop near the ATP-binding site of the kinase domain across all three PIP4K2 isoforms. This binding leads to the irreversible inhibition of their enzyme activity [14]. Therefore, the combination of venetoclax and THZ-P1-2 was tested in multiple cellular models of AML. In Kasumi-1, NB4, and U-937 cells, THZ-P1-2 showed potentiating effects on the reduction in cell viability caused by venetoclax (Figure 3A). These findings were confirmed through the analysis of apoptosis using flow cytometry (Figure 3B) and through the detection of molecular markers of cell death (increase in cleaved PARP1 and expression of γH2AX; Figure 3C).

### 2.4. Differential Gene Expression upon THZ-P1-2 and/or Venetoclax Exposure in Kasumi-1 Cells

**Figure 4:**
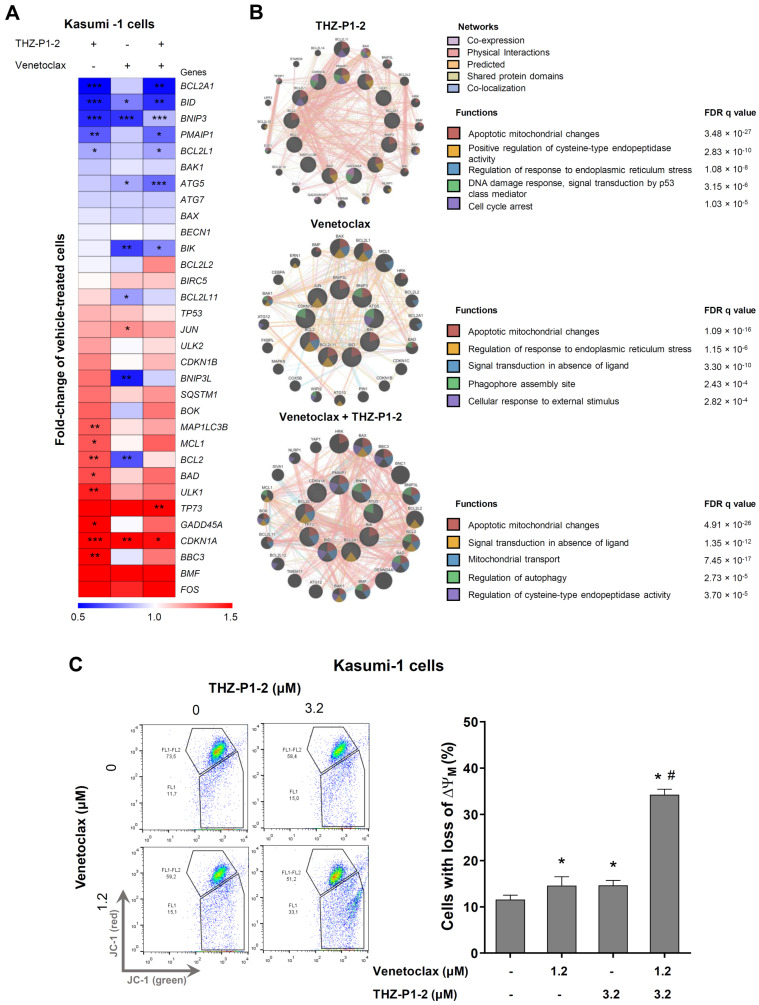
Effects of THZ-P1-2 alone or in combination with venetoclax on the gene expression profile of Kasumi-1 cells. (**A**) Heatmap for the gene expression analysis in Kasumi-1 cells treated with vehicle, THZ-P1-2 (3.2 µM), and/or venetoclax (1.2 µM) for 24 h. The data represent the fold change of the vehicle-treated cells, and the downregulated and upregulated genes are shown in blue and red, respectively. The fold-change (FC), standard deviation (SD), and *p*-values are indicated, with * *p* < 0.05, ** *p* < 0.01, *** *p* < 0.001, using Student t-test. (**B**) The network for the genes modulated with THZ-P1-2 and/or venetoclax was constructed using the GeneMANIA database (https://genemania.org/ (accessed on 2 August 2023)). The genes significantly modulated are illustrated as crosshatched circles; the interacting genes included by modeling the software are indicated using circles without crosshatching. The main biological interactions and the associated functions are described. FDR, false discover rate. (**C**) The mitochondrial membrane potential (ΔΨM) analysis was evaluated using the JC-1 staining method and flow cytometry. Kasumi-1 cells were treated with vehicle, THZ-P1-2 (3.2 μM), and/or venetoclax (1.2 μM) for 24 h. Representative dot plots are shown for each condition; the gate FL-2 contains cells with intact mitochondria and the gate FL-2/FL-1 contains cells with damaged mitochondria. The bar graphs represent the mean ± SD of at least five independent experiments and the *p* values are indicated as follows: * *p* < 0.05 treatment versus vehicle and ^#^ *p* < 0.05 monotherapy versus combined therapy; ANOVA test and Bonferroni post-test.

Finally, to better understand the molecular events triggered by THZ-P1-2 in the presence or absence of venetoclax, a panel of genes associated with cell cycle progression, apoptosis, DNA damage, and autophagy were evaluated. In the Kasumi-1 cells, a total of thirteen and nine genes were modulated using THZ-P1-2 and venetoclax monotherapies, respectively. The combination of THZ-P1-2 and venetoclax modulated nine genes, highlighting the upregulation of *TAp73* as a target significantly modulated only in the combined treatment (Figure 4A and Table S1). In a network analysis, the genes differentially expressed upon THZ-P1-2 treatment were associated with apoptotic mitochondrial changes, a positive regulation of cysteine-type endopeptidase activity, a regulation of the response to endoplasmic reticulum stress, DNA damage response (signal transduction by p53 class mediator), and cell cycle arrest. The venetoclax treatment was associated with apoptotic mitochondrial changes, a regulation of the response to endoplasmic reticulum stress, signal transduction in the absence of ligand, phagophore assembly site, and a cellular response to external stimulus. The combined treatment, THZ-P1-2 plus venetoclax, was associated with apoptotic mitochondrial changes, signal transduction in the absence of ligand, mitochondrial transport, regulation of autophagy, and the regulation of cysteine-type endopeptidase activity (Figure 4B). Corroborating our molecular findings, the combination of THZ-P1-2 and venetoclax elicited a more pronounced degree of mitochondrial damage when compared to the monotherapies (Figure 4C).

## 3. Discussion

Here, we characterized PIP4K2 expression in normal and malignant myeloid cells. The high expression of *PIP4K2A* found in erythroid cells agrees with the initial description of this protein in the literature, reporting its abundance in erythrocytes [15], and with more recent findings showing that it participates in the process of erythroid differentiation and hemoglobin production [16,17,18]. Along the same line, we described high levels of PIP4K2A in myeloid cell lines committed to the erythro-megakaryocytic compartment (K-562, KU812, SET2, and HEL). Regarding *PIP4K2B*, our data suggested that it is highly expressed in hematopoietic stem cells, which deserve future study. In primary cells from AML patients, the highest levels of *PIP4K2A* and *PIP4K2C* were observed in unfavorable cytogenetic risk groups, which agrees with previous studies by our group in two independent cohorts [10,19]. Of note, both *PIP4K2A* and *PIP4K2C* have been previously associated with unfavorable clinical outcomes in AML [10].

The analysis of the association between PIP4K2 genes and sensitivity to antineoplastic drugs in ex vivo assays in AML identified targets of clinical importance in hematological malignancies, including ABL1, EGFR, VEGFR, AKT, ERK, BCL2, and FLT3 [20,21,22,23,24]. In the present study, we chose to evaluate the combination of THZ-P1-2 and venetoclax in different AML models. Venetoclax is a selective BCL2 inhibitor that has gained prominence in the treatment of lymphoid and myeloid malignancies, and its use has become the standard of care for patients with AML ineligible for intensive chemotherapy, being active in all spectra of risk groups, inducing a complete hematological remission with complete (CHR) or incomplete blood count recovery (Cri) in more than 65% of patients [25,26,27,28,29,30]. In addition, the rate of deaths in the first 30 or 60 days was lower than that reported with intensive chemotherapy; this observation represents an advantage in the context of constraint resources where these values exceed 20% [31]. Nevertheless, the median duration of the response was 17.8 months in the Vial A trial [25]. In the present study, THZ-P1-2 potentiates apoptosis induced using venetoclax in multiple AML models with different degrees of sensitivity to the drug. In a previous preliminary study with OCI-AML3 cells, similar results were observed, corroborating the current experimental findings [6].

The molecular analysis indicates that the combination of THZ-P1-2 and venetoclax induces increased levels of PARP1 and γH2AX, markers of apoptosis and DNA damage [32,33]. The *TP73* gene was significantly upregulated only in the combined treatment. TP73 was the first TP53 homologous gene described [34]. The *TP73* transcript evaluated in this study corresponds to the *TAp73* isoform, which functionally performs functions similar to the p53 protein [35]. Furthermore, the combined treatment of THZ-P1-2 and venetoclax suppressed antiapoptotic genes, such as *MCL1* and *BCL2*, induced in THZ-P1-2 monotherapy, which could also explain the better cell death rates. The *MCL1* gene, a member of the BCL2 family, deserves attention as its relevance has been consolidated by multiple research groups as a potent mechanism of resistance to venetoclax [36,37,38]. The treatment with THZ-P1-2 alone or combination therapy suppressed the expression of *BCL2A1*, another antiapoptotic member of the BCL2 family associated with venetoclax resistance in AML [39,40]. Our findings suggest that, among other effects, THZ-P1-2 may act as a mitocan, which is a diverse group of anti-cancer compounds that target mitochondria. They disrupt energy production leading to the enhanced generation of reactive oxygen species along with the activation of the intrinsic pathway of apoptosis [41]. In the context of overcoming resistance to venetoclax, or improving its effects in AML, preclinical studies have shown promising results in studies analyzing molecules targeting mitochondrial respiration or structure, which include those disrupting the mitochondrial electron transport chain, mitochondrial translation, mitochondrial DNA replication, or mitochondrial protease ClpP [42].

## 4. Materials and Methods

### 4.1. Expression Data in Normal and Malignant Hematopoietic Cells

*PIP4K2A* (probe 205570_at), *PIP4K2B* (probe 201080_at), and *PIP4K2C* (probe 218942_at) mRNA expression data from the different hematopoietic cell populations were obtained from the GSE24759 dataset using the GEO2R platform (https://www.ncbi.nlm.nih.gov/geo/geo2r (accessed on 2 August 2023)). The gene’s expression values were obtained from cDNA microarray experiments using the Affymetrix HUG133 plus 2.0 arrays system and the data were crossed using specific identification numbers. Graphical visualizations of *PIP4K2A*, *PIP4K2B*, and *PIP4K2C* mRNA levels in different molecular subtypes of AML (TCGA AML study [43]) were obtained from BloodSpot (https://servers.binf.ku.dk/bloodspot/ (accessed on 2 August 2023)).

### 4.2. Cell Lines and Inhibitors

OCI-AML3, Kasumi-1, MOLM-13, and MV4-11 cells were obtained from Deutsche Sammlung von Mikroorganismen and Zellkulturen (DSMZ, Braunschweig, Germany). THP-1 and HL-60 cells were obtained from the American Type Culture Collection (ATCC, Manassas, VA, USA). NB4 cells were kindly provided by Prof. Pier Paolo Pandolfi (Beth Israel Deaconess Medical Center, Havard Medical School, Boston, MA, USA). U-937, K-562, KU812, and HEL cells were kindly provided by Prof. Sara Teresinha Olalla Saad (University of Campinas, Campinas, Brazil). SET-2 cells were kindly provided by Prof. Fabíola Attié de Castro (University of São Paulo, Ribeirão Preto, Brazil). Cells were cultured in appropriate media (RPMI-1640, IMDM, or alpha-MEM), supplemented with 10 or 20% of fetal bovine serum (FBS) according to the ATCC’s and DSMZ’s recommendations, plus 1% of penicillin/streptomycin, and maintained at 5% of CO_2_ and 37°C. THZ-P1-2 was obtained from MedChemExpress (Monmouth Junction, NJ, USA) and diluted to 25 mM in dimethyl sulfoxide (Me_2_SO_4_; DMSO). Venetoclax (ABT-199) was obtained from TargetMol (Target Molecule Corp. Boston, MA, USA) and diluted to a 50 mM stock solution in DMSO.

### 4.3. Primary Cells

CD34^+^ progenitors were separated on MIDI-MACS immunoaffinity columns (Miltenyi Biotec, Auburn, CA, USA) from the peripheral blood of a healthy donor. Informed consent was obtained from the healthy donor and the Ethics Committee of the Institute of Biomedical Sciences of the University of São Paulo approved this study (Protocol number: 4423074; CAAE: 39510920.1.0000.5467).

### 4.4. Quantitative RT-PCR (qRT-PCR)

RNA was extracted using the TRIzol reagent (Thermo Fisher Scientific, Cleveland, OH, USA). cDNA was synthesized from 1 μg of RNA using a High-Capacity cDNA Reverse Transcription Kit (Thermo Fisher Scientific). Quantitative PCR (qPCR) was performed using a QuantStudio 3 Real-Time PCR System in conjunction with a SybrGreen System (Thermo Fisher Scientific) to assess the expression of PIP4K2, cell cycle-, apoptosis-, DNA-damage-, and autophagy-related genes (Table S2). *HPRT1* and *ACTB* were used as reference genes. Relative quantification values were calculated using the 2^-ΔΔCT^ equation [44]. A negative ‘No Template Control’ was included for each primer pair. The data were illustrated using the multiple experiment viewer (MeV) 4.9.0 software [45].

### 4.5. Western Blotting

Protein extraction was performed using a buffer containing 100 mM of Tris (pH 7.6), 1% of Triton X-100, 2 mM of PMSF, 10 mM of Na_3_VO_4_, 100 mM of NaF, 10 mM of Na_4_P_2_O_7_, and 4 mM of EDTA. Equal amounts of protein (30 μg) from the samples were subsequently subjected to SDS-PAGE in an electrophoresis device, followed by the electrotransfer of the proteins to nitrocellulose membranes. The membranes were blocked with 5% of non-fat dry milk and incubated with specific primary antibodies diluted in a blocking buffer, followed by the secondary antibodies conjugated to horseradish peroxidase (HRP). A Western blot analysis was performed using a SuperSignal^TM^ West Dura Extended Duration substrate system (Thermo Fisher Scientific) and G: BOX Chemi XX6 gel document system (Syngene Cambridge, Cambridge, UK). Antibodies directed against PIP4K2A (#5527), PIP4K2B (#9694), PARP1 (#9542), γH2AX (#9718), and α-tubulin (#2144) were obtained from Cell Signaling Technology (Danvers, MA, USA). Antibody directed against PIP4K2C (PA5-15289) were obtained from Thermo Fisher Scientific. Cropped gels retained important bands, but whole gel images are available in the Supplementary Materials.

### 4.6. Drug Sensitivity Prediction

To explore the correlation between drug response and the *PIP4K2A*, *PIP4K2B*, and *PIP4K2C* expression, the AUC values resulting from 165 drugs tested ex vivo assays with a Beat AML study (n = 520) were used [11,12]. For a better interpretation of the results obtained, the molecular targets (proteins/genes) of the identified drugs were listed and analyzed using Venn diagrams (http://bioinformatics.psb.ugent.be/ (accessed on 2 August 2023)).

### 4.7. Cell Viability Assay

In total, 2 × 10^4^ cells per well were seeded in a 96-well plate in the appropriate medium in the presence of vehicle or different concentrations of THZ-P1-2 (1.6, 3.2, or 6.4 µM) alone or in combination with venetoclax (0.3, 0.6, 1.2, or 2.5 µM) for 48 h. Next, 10 μL of methylthiazoletetrazolium (MTT, Sigma-Aldrich, St. Louis, MO, USA) solution (5 mg/mL) was added and incubated at 37 °C and 5% of CO_2_ for 4 h. The reaction was stopped using 100 μL of 0.1 N HCl in anhydrous isopropanol. Cell viability was evaluated by measuring the absorbance at 570 nm. The data were illustrated using the multiple experiment viewer (MeV) 4.9.0 software [45].

### 4.8. Apoptosis Assay

In total, 1 × 10^5^ cells per well were seeded in a 24-well plate in an FBS-supplemented medium in the presence of vehicle or THZ-P1-2 (3.2 or 6.4 μM) alonea or in combination with venetoclax (0.6, 1.2, or 2.5 μM) for 48h. Next, the cells were washed with ice-cold phosphate-buffered saline (PBS) and resuspended in a binding buffer containing 1 μg/mL of propidium iodide (PI) and 1 μg/mL of APC-labeled annexin V (BD Pharmingen, San Diego, CA, USA). All the specimens were analyzed using flow cytometry (FACSCalibur) after incubation for 15 min at room temperature in a light-protected area. Ten thousand events were recorded for each sample.

### 4.9. Mitochondrial Membrane Potential Evaluation

In total, 2 × 10^5^ Kasumi-1 cells per well were seeded in a 24-well plate in 20% of FBS RPMI in the presence of vehicle, THZ-P1-2 (3.2 μM), and/or venetoclax (1.2 μM) for 24 h. Cells were then washed with PBS and resuspended in a buffer containing 5 μg/mL of JC-1 (BD), and 10,000 events were acquired using flow cytometry (FACSCalibur) after incubation for 15 min at 37°C and 5% of CO_2_ in a light-protected area. The events were analyzed using the FlowJo software v.X.0.7 (Treestar, Inc., Mesa, AZ, USA).

### 4.10. Statistical Analysis

Statistical analyses were performed using GraphPad Prism 8 v.8.0.2 (GraphPad Software, Inc. San Diego, CA, USA). ANOVA, Bonferroni post-test, and Student’s t-test were used for comparisons as appropriate. Gene networks were constructed using the GeneMANIA database (https://genemania.org/ (accessed on 2 August 2023)). All the *p*-values were two-sided with a significance level of 5%.

## 5. Conclusions

Our study characterized the expression of PIP4K2 in the myeloid compartment of hematopoietic cells, as well as in different molecular subsets of AML. The identification of the correlation between the expression of PIP4K2 and the response to antineoplastic agents in ex vivo assays in AML exposed vulnerabilities that can be exploited in combined therapies, which could result in better therapeutic responses.

## Data Availability

The datasets used and/or analyzed during the current study are available from the corresponding author upon reasonable request.

## Supplementary Materials

The following supporting information can be downloaded at: https://www.mdpi.com/article/10.3390/ijms242316899/s1.

## References

[1] Short N.J., Rytting M.E., Cortes J.E. (2018). Acute myeloid leukaemia. Lancet.

[2] Dohner H., Weisdorf D.J., Bloomfield C.D. (2015). Acute Myeloid Leukemia. N. Engl. J. Med..

[3] Liersch R., Muller-Tidow C., Berdel W.E., Krug U. (2014). Prognostic factors for acute myeloid leukaemia in adults—Biological significance and clinical use. Br. J. Haematol..

[4] Kayser S., Levis M.J. (2022). Updates on targeted therapies for acute myeloid leukaemia. Br. J. Haematol..

[5] DiNardo C.D., Erba H.P., Freeman S.D., Wei A.H. (2023). Acute myeloid leukaemia. Lancet.

[6] Lima K., Pereira-Martins D.A., de Miranda L.B.L., Coelho-Silva J.L., Leandro G.D.S., Weinhauser I., Cavaglieri R.C., Leal A.M., da Silva W.F., Lange A. (2022). The PIP4K2 inhibitor THZ-P1-2 exhibits antileukemia activity by disruption of mitochondrial homeostasis and autophagy. Blood Cancer J..

[7] Arora G.K., Palamiuc L., Emerling B.M. (2022). Expanding role of PI5P4Ks in cancer: A promising druggable target. FEBS Lett..

[8] Fiume R., Stijf-Bultsma Y., Shah Z.H., Keune W.J., Jones D.R., Jude J.G., Divecha N. (2015). PIP4K and the role of nuclear phosphoinositides in tumour suppression. Biochim. Biophys. Acta.

[9] Jude J.G., Spencer G.J., Huang X., Somerville T.D.D., Jones D.R., Divecha N., Somervaille T.C.P. (2015). A targeted knockdown screen of genes coding for phosphoinositide modulators identifies PIP4K2A as required for acute myeloid leukemia cell proliferation and survival. Oncogene.

[10] Lima K., Coelho-Silva J.L., Kinker G.S., Pereira-Martins D.A., Traina F., Fernandes P., Markus R.P., Lucena-Araujo A.R., Machado-Neto J.A. (2019). PIP4K2A and PIP4K2C transcript levels are associated with cytogenetic risk and survival outcomes in acute myeloid leukemia. Cancer Genet..

[11] Tyner J.W., Tognon C.E., Bottomly D., Wilmot B., Kurtz S.E., Savage S.L., Long N., Schultz A.R., Traer E., Abel M. (2018). Functional genomic landscape of acute myeloid leukaemia. Nature.

[12] Bottomly D., Long N., Schultz A.R., Kurtz S.E., Tognon C.E., Johnson K., Abel M., Agarwal A., Avaylon S., Benton E. (2022). Integrative analysis of drug response and clinical outcome in acute myeloid leukemia. Cancer Cell.

[13] Bruzzese A., Martino E.A., Mendicino F., Lucia E., Olivito V., Neri A., Morabito F., Vigna E., Gentile M. (2023). Venetoclax in acute myeloid leukemia. Expert Opin. Investig. Drugs.

[14] Sivakumaren S.C., Shim H., Zhang T., Ferguson F.M., Lundquist M.R., Browne C.M., Seo H.S., Paddock M.N., Manz T.D., Jiang B. (2020). Targeting the PI5P4K Lipid Kinase Family in Cancer Using Covalent Inhibitors. Cell Chem. Biol..

[15] Ling L.E., Schulz J.T., Cantley L.C. (1989). Characterization and purification of membrane-associated phosphatidylinositol-4-phosphate kinase from human red blood cells. J. Biol. Chem..

[16] Wenning M.R., Mello M.P., Andrade T.G., Lanaro C., Albuquerque D.M., Saad S.T., Costa F.F., Sonati M.F. (2009). PIP4KIIA and beta-globin: Transcripts differentially expressed in reticulocytes and associated with high levels of Hb H in two siblings with Hb H disease. Eur. J. Haematol..

[17] Zaccariotto T.R., Lanaro C., Albuquerque D.M., Santos M.N., Bezerra M.A., Cunha F.G., Lorand-Metze I., Araujo A.S., Costa F.F., Sonati M.F. (2012). Expression profiles of phosphatidylinositol phosphate kinase genes during normal human in vitro erythropoiesis. Genet. Mol. Res..

[18] Zhang Y., Xie H., Liang G., Qin Y., Wei X., Ning S., Liang Y., Liang X., Xie Y., Lin Z. (2023). A novel gain-of-function PIP4K2A mutation elevates the expression of beta-globin and aggravates the severity of alpha-thalassemia. Br. J. Haematol..

[19] Zhang S., Li Z., Yan X., Bao L., Deng Y., Zeng F., Wang P., Zhu J., Yin D., Liao F. (2018). Regulatory Network and Prognostic Effect Investigation of PIP4K2A in Leukemia and Solid Cancers. Front. Genet..

[20] Foa R., Bassan R., Vitale A., Elia L., Piciocchi A., Puzzolo M.C., Canichella M., Viero P., Ferrara F., Lunghi M. (2020). Dasatinib-Blinatumomab for Ph-Positive Acute Lymphoblastic Leukemia in Adults. N. Engl. J. Med..

[21] Hughes T.P., Mauro M.J., Cortes J.E., Minami H., Rea D., DeAngelo D.J., Breccia M., Goh Y.T., Talpaz M., Hochhaus A. (2019). Asciminib in Chronic Myeloid Leukemia after ABL Kinase Inhibitor Failure. N. Engl. J. Med..

[22] Santinelli E., Pascale M.R., Xie Z., Badar T., Stahl M.F., Bewersdorf J.P., Gurnari C., Zeidan A.M. (2023). Targeting apoptosis dysregulation in myeloid malignancies*—*The promise of a therapeutic revolution. Blood Rev..

[23] Kampen K.R., Ter Elst A., de Bont E.S. (2013). Vascular endothelial growth factor signaling in acute myeloid leukemia. Cell Mol. Life Sci..

[24] Short N.J., Nguyen D., Ravandi F. (2023). Treatment of older adults with FLT3-mutated AML: Emerging paradigms and the role of frontline FLT3 inhibitors. Blood Cancer J..

[25] DiNardo C.D., Jonas B.A., Pullarkat V., Thirman M.J., Garcia J.S., Wei A.H., Konopleva M., Dohner H., Letai A., Fenaux P. (2020). Azacitidine and Venetoclax in Previously Untreated Acute Myeloid Leukemia. N. Engl. J. Med..

[26] DiNardo C.D., Pratz K., Pullarkat V., Jonas B.A., Arellano M., Becker P.S., Frankfurt O., Konopleva M., Wei A.H., Kantarjian H.M. (2019). Venetoclax combined with decitabine or azacitidine in treatment-naive, elderly patients with acute myeloid leukemia. Blood.

[27] Pollyea D.A., Amaya M., Strati P., Konopleva M.Y. (2019). Venetoclax for AML: Changing the treatment paradigm. Blood Adv..

[28] Griffioen M.S., de Leeuw D.C., Janssen J., Smit L. (2022). Targeting Acute Myeloid Leukemia with Venetoclax; Biomarkers for Sensitivity and Rationale for Venetoclax-Based Combination Therapies. Cancers.

[29] Hu M., Li W., Zhang Y., Liang C., Tan J., Wang Y. (2023). Venetoclax in adult acute myeloid leukemia. Biomed. Pharmacother..

[30] Wei A.H., Roberts A.W. (2023). BCL2 Inhibition: A New Paradigm for the Treatment of AML and Beyond. Hemasphere.

[31] Mendes F.R., da Silva W.F., da Costa Bandeira de Melo R., Silveira D.R.A., Velloso E., Rocha V., Rego E.M. (2022). Predictive factors associated with induction-related death in acute myeloid leukemia in a resource-constrained setting. Ann. Hematol..

[32] Kaufmann S.H., Desnoyers S., Ottaviano Y., Davidson N.E., Poirier G.G. (1993). Specific proteolytic cleavage of poly(ADP-ribose) polymerase: An early marker of chemotherapy-induced apoptosis. Cancer Res..

[33] Mah L.J., El-Osta A., Karagiannis T.C. (2010). gammaH2AX: A sensitive molecular marker of DNA damage and repair. Leukemia.

[34] Kaghad M., Bonnet H., Yang A., Creancier L., Biscan J.C., Valent A., Minty A., Chalon P., Lelias J.M., Dumont X. (1997). Monoallelically expressed gene related to p53 at 1p36, a region frequently deleted in neuroblastoma and other human cancers. Cell.

[35] Jost C.A., Marin M.C., Kaelin W.G. (1997). p73 is a simian [correction of human] p53-related protein that can induce apoptosis. Nature.

[36] Ramsey H.E., Fischer M.A., Lee T., Gorska A.E., Arrate M.P., Fuller L., Boyd K.L., Strickland S.A., Sensintaffar J., Hogdal L.J. (2018). A Novel MCL1 Inhibitor Combined with Venetoclax Rescues Venetoclax-Resistant Acute Myelogenous Leukemia. Cancer Discov..

[37] Pan R., Hogdal L.J., Benito J.M., Bucci D., Han L., Borthakur G., Cortes J., DeAngelo D.J., Debose L., Mu H. (2014). Selective BCL-2 inhibition by ABT-199 causes on-target cell death in acute myeloid leukemia. Cancer Discov..

[38] Zhang Q., Riley-Gillis B., Han L., Jia Y., Lodi A., Zhang H., Ganesan S., Pan R., Konoplev S.N., Sweeney S.R. (2022). Activation of RAS/MAPK pathway confers MCL-1 mediated acquired resistance to BCL-2 inhibitor venetoclax in acute myeloid leukemia. Signal Transduct. Target. Ther..

[39] Zhang H., Nakauchi Y., Kohnke T., Stafford M., Bottomly D., Thomas R., Wilmot B., McWeeney S.K., Majeti R., Tyner J.W. (2020). Integrated analysis of patient samples identifies biomarkers for venetoclax efficacy and combination strategies in acute myeloid leukemia. Nat. Cancer.

[40] Bisaillon R., Moison C., Thiollier C., Krosl J., Bordeleau M.E., Lehnertz B., Lavallee V.P., MacRae T., Mayotte N., Labelle C. (2020). Genetic characterization of ABT-199 sensitivity in human AML. Leukemia.

[41] Panina S.B., Baran N., Brasil da Costa F.H., Konopleva M., Kirienko N.V. (2019). A mechanism for increased sensitivity of acute myeloid leukemia to mitotoxic drugs. Cell Death Dis..

[42] Garciaz S., Saillard C., Hicheri Y., Hospital M.A., Vey N. (2021). Venetoclax in Acute Myeloid Leukemia: Molecular Basis, Evidences for Preclinical and Clinical Efficacy and Strategies to Target Resistance. Cancers.

[43] Ley T.J., Miller C., Ding L., Raphael B.J., Mungall A.J., Robertson A., Hoadley K., Triche T.J., Jr Laird P.W., Baty J.D. (2013). Genomic and epigenomic landscapes of adult de novo acute myeloid leukemia. N. Engl. J. Med..

[44] Livak K.J., Schmittgen T.D. (2001). Analysis of relative gene expression data using real-time quantitative PCR and the 2(-Delta Delta C(T)) Method. Methods.

[45] Saeed A.I., Sharov V., White J., Li J., Liang W., Bhagabati N., Braisted J., Klapa M., Currier T., Thiagarajan M. (2003). TM4: A free, open-source system for microarray data management and analysis. Biotechniques.

