# Supplementary material for: Pharmacological Inhibition of PIP4K2 Potentiates Venetoclax-Induced Apoptosis in Acute Myeloid Leukemia
Source: Int J Mol Sci. 2023 Nov 29;24(23):16899. doi: 10.3390/ijms242316899 (PMC10706459; doi:10.3390/ijms242316899)
Supplement: Supplementary file 1 [file ijms-24-16899-s001.zip › Table S1.pdf]

**Table S1. Effects of THZ-P1-2 alone or in combination with venetoclax on gene expression profile of Kasumi-1 cells.**

| Genes | THZ-P1-2 | | | Venetoclax | | | THZ-P1-2 plus venetoclax | | |
|---|---|---|---|---|---|---|---|---|---|
| | FC[1] | SD | *p*[2] | FC[1] | SD | *p*[2] | FC[1] | SD | *p*[2] |
| *BCL2A1* | 0,30 | 0,22 | *** | 0,90 | 0,20 | n.s. | 0,52 | 0,16 | ** |
| *BID* | 0,55 | 0,11 | *** | 0,75 | 0,19 | * | 0,59 | 0,10 | ** |
| *BNIP3* | 0,58 | 0,15 | *** | 0,60 | 0,15 | *** | 0,86 | 0,19 | *** |
| *PMAIP1* | 0,68 | 0,11 | ** | 0,90 | 0,19 | n.s. | 0,70 | 0,11 | * |
| *BCL2L1* | 0,83 | 0,11 | * | 0,90 | 0,10 | n.s. | 0,82 | 0,08 | * |
| *BAK1* | 0,89 | 0,12 | n.s. | 0,95 | 0,22 | n.s. | 0,92 | 0,19 | n.s. |
| *ATG5* | 0,90 | 0,09 | n.s. | 0,83 | 0,07 | * | 0,71 | 0,08 | *** |
| *ATG7* | 0,91 | 0,12 | n.s. | 0,91 | 0,30 | n.s. | 0,90 | 0,24 | n.s. |
| *BAX* | 0,95 | 0,11 | n.s. | 0,91 | 0,15 | n.s. | 0,92 | 0,10 | n.s. |
| *BECN1* | 0,97 | 0,24 | n.s. | 1,00 | 0,40 | n.s. | 0,97 | 0,29 | n.s. |
| *BIK* | 0,97 | 0,11 | n.s. | 0,62 | 0,18 | ** | 0,77 | 0,10 | * |
| *BCL2L2* | 0,98 | 0,11 | n.s. | 0,93 | 0,27 | n.s. | 1,23 | 0,39 | n.s. |
| *BIRC5* | 1,01 | 1,01 | n.s. | 1,11 | 0,17 | n.s. | 1,11 | 0,16 | n.s. |
| *BCL2L11* | 1,08 | 0,11 | n.s. | 0,81 | 0,09 | * | 0,92 | 0,08 | n.s. |
| *TP53* | 1,15 | 0,08 | n.s. | 1,12 | 0,10 | n.s. | 1,05 | 0,11 | n.s. |
| *JUN* | 1,17 | 0,12 | n.s. | 1,22 | 0,17 | * | 1,18 | 0,07 | n.s. |
| *ULK2* | 1,19 | 0,24 | n.s. | 0,99 | 0,29 | n.s. | 1,07 | 0,21 | n.s. |
| *CDKN1B* | 1,24 | 0,20 | n.s. | 1,01 | 0,22 | n.s. | 1,14 | 0,08 | n.s. |
| *BNIP3L* | 1,28 | 0,25 | n.s. | 0,55 | 0,09 | ** | 0,91 | 0,10 | n.s. |
| *SQSTM1* | 1,28 | 0,12 | n.s. | 1,09 | 0,27 | n.s. | 1,21 | 0,22 | n.s. |
| *BOK* | 1,31 | 0,18 | n.s. | 0,89 | 0,14 | n.s. | 1,27 | 0,24 | n.s. |
| *MAP1L3B* | 1,32 | 0,07 | ** | 1,06 | 0,21 | n.s. | 1,18 | 0,06 | n.s. |
| *MCL1* | 1,33 | 0,22 | * | 1,02 | 0,17 | n.s. | 1,31 | 0,18 | n.s. |
| *BCL2* | 1,34 | 0,13 | ** | 0,66 | 0,06 | ** | 1,06 | 1,06 | n.s. |
| *BAD* | 1,35 | 0,16 | * | 1,09 | 0,31 | n.s. | 1,29 | 0,09 | n.s. |
| *ULK1* | 1,44 | 0,18 | ** | 1,18 | 0,18 | n.s. | 1,27 | 0,21 | n.s. |
| *TP73* | 1,54 | 0,42 | n.s. | 1,52 | 0,35 | n.s. | 2,13 | 0,65 | ** |
| *GADD45A* | 1,71 | 0,13 | * | 0,99 | 0,49 | n.s. | 1,30 | 0,43 | n.s. |
| *CDKN1A* | 1,78 | 0,36 | *** | 1,59 | 0,13 | ** | 1,48 | 0,06 | * |
| *BBC3* | 1,86 | 0,30 | ** | 0,95 | 0,40 | n.s. | 1,27 | 0,09 | n.s. |
| *BMF* | 2,21 | 2,09 | n.s. | 2,48 | 1,16 | n.s. | 2,11 | 1,36 | n.s. |
| *FOS* | 4,19 | 2,32 | n.s. | 1,43 | 0,50 | n.s. | 4,40 | 4,97 | n.s. |

Abbreviations: FC, fold-change; SD, standard deviation; n.s., non-significant.

[1] Fold-change to vehicle-treated cells.

[2] Student t test; *$p < 0.05$, **$p < 0.01$, ***$p < 0.001$.
