# Supplementary material for: Pharmacological Inhibition of PIP4K2 Potentiates Venetoclax-Induced Apoptosis in Acute Myeloid Leukemia
Source: Int J Mol Sci. 2023 Nov 29;24(23):16899. doi: 10.3390/ijms242316899 (PMC10706459; doi:10.3390/ijms242316899)
Supplement: Supplementary file 1 [file ijms-24-16899-s001.zip › Table S2.pdf]

Table S2. Primer sequences and concentrations for quantitative PCR.

Gene[1]	Sequence	Concentration
ACTB	FW: AGGCCAACCGCGAGAAG<br>RV: ACAGCCTGGATAGCAACGTACA	150 nM
ATG5	FW: GGGCCATCAATCGGAAAC<br>RV: AGCCACAGGACGAAACAG	300 nM
ATG7	FW: CGTTGCCCACAGCATCATCTTC<br>RV: TCCCATGCCTCCTTTCTGGTTC	300 nM
BAD	FW: CACCAGCAGGAGCAGCCAAC<br>RV: CGACTCCGGATCTCCACAGC	300 nM
BAK1	FW: TGAGTACTTCACCAAGATTGCCA<br>RV: AGTCAGGCCATGCTGGTAGAC	300 nM
BAX	FW: GAGCTGCAGAGGATGATTGC<br>RV: CAGCTGCCACTCGGAAAA	300 nM
BBC3	FW: GACCTCAACGCACAGTACGAG<br>RV: AGGAGTCCCATGATGAGATTGT	300 nM
BCL2	FW: ATGTGTGTGGAGAGCGTCAA<br>RV: ACAGTTCCACAAAGGCATCC	300 nM
BCL2A1	FW: TTACAGGCTGGCTCAGGACT<br>RV: AGCACTCTGGACGTTTTGCT	300 nM
BCL2L1	FW: CTTGGATGGCCACTTACCTGAA<br>RV: GCTGCTGCATTGTTCCCATA	300 nM
BCL2L11	FW: ATGTCTGACTCTGACTCTCG<br>RV: CCTTGTGGCTCTGTCTGTAG	300 nM
BCL2L2	FW: GCGGAGTTCACAGCTCTATAC<br>RV: AAAAGGCCCCTACAGTTACCA	300 nM
BECN1	FW: TCTGAAGAGGACCTGGACCCT<br>RV: GGCTCACGTCCATCTCGTC	300 nM
BID	FW: ATGGACCGTAGCATCCCTCC<br>RV: GTAGGTGCGTAGGTTCTGGT	300 nM
BIK	FW: TCTGAAGAGGACCTGGACCCT<br>RV: GGCTCACGTCCATCTCGTC	300 nM
BIRC5	FW: GCCCAGTCTTTCTTCTGCTTCA<br>RV: GACCTTTCTCCGCAGTTTCCTC	300 nM
BMF	FW: CCTCCTTCCCAATCGAGTCTG<br>RV: TCCATCTCTCCTGGGTGACT	300 nM
BNIP3	FW: ATATGGGATTGGTCAAGTCGG<br>RV: CGCTCGTGTTCCTCATGCT	300 nM
BNIP3L	FW: ACACCAGCAGGGACCATAGC<br>RV: TTTCTTCAAAGCCTCGACTTCC	300 nM
BOK	FW: GCGATGAGCTGGAGATGATCC<br>RV: CTGCAGAGAAGATGTGGCCA	300 nM
CDKN1A	FW: TGTCACTGTCTTGTACCCTTGT<br>RV: GCCGGCGTTTGGAGTGGTAG	300 nM
CDKN1B	FW: ACTCTGAGGACACGCATTTGGT<br>RV: TCTGTTCTGTTGGCTCTTTTGTT	300 nM
FOS	FW: AGAATCCGAAGGGAAAGGAA<br>RV: CTTCTCCTTCAGCAGGTTGG	300 nM
GADD45A	FW: AAGGATGGATAAGGTGGGG<br>RV: CTGGATCAGGGTGAAGTGG	300 nM

HPRT1	FW: GAACGTCTTGCTCGAGATGTGA<br>RV: TCCAGCAGGTCAGCAAAGAAT	150 nM
JUN	FW: CAGGTGGCACAGCTTAAACA<br>RV: GTTTGCAACTGCTGCGTTAG	300 nM
MAP1LC3B	FW: AAGGCGCTTACAGCTCAATG<br>RV: CTGGGAGGCATAGACCATGT	300 nM
MCL1	FW: GTAATAACACCAGTACGGACGG<br>RV: TCCCGAAGGTACCGAGAGAT	300 nM
PMAIP1	FW: CGCGCAAGAACGCTCAACC<br>RV: CACACTCGACTTCCAGCTCTGCT	300 nM
SQSTM1	FW: TGAGGAACAGATGGAGTCGGATAA<br>RV: GGGACTGGAGTTCACCTGTAGACG	300 nM
TP53	FW: GGCGCACAGAGGAAGAGAAT<br>RV: GGAGAGGAGCTGGTGTTGTTG	300 nM
TP73	FW: GCACCACGTTTGAGCACCTCT<br>RV: GCAGATTGAACTGGGCCATGA	300 nM
ULK1	FW: CAGACAGCCTGATGTGCAGT<br>RV: CAGGGTGGGGATGGAGAT	300 nM
ULK2	FW: GACGAGTAACCAAGGCTAACAG<br>RV: GGCTCTCCTACTAAGACCACAG	300 nM

[1]Genes are reported according to Human Genome Organisation (HUGO) Gene Nomenclature Committee (HGNC).

Abbreviations: FW, forward; RV, reverse.
