# Supplementary figures and images for: Pharmacological Inhibition of PIP4K2 Potentiates Venetoclax-Induced Apoptosis in Acute Myeloid Leukemia
Source: Int J Mol Sci. 2023 Nov 29;24(23):16899. doi: 10.3390/ijms242316899 (PMC10706459; doi:10.3390/ijms242316899)

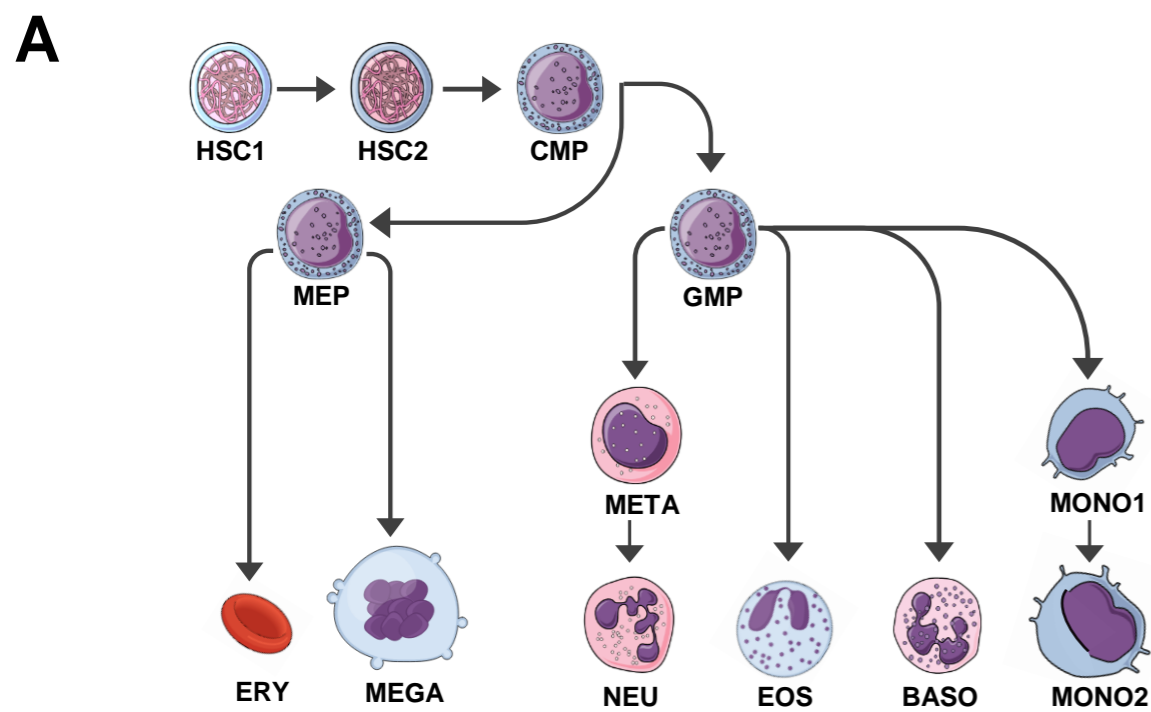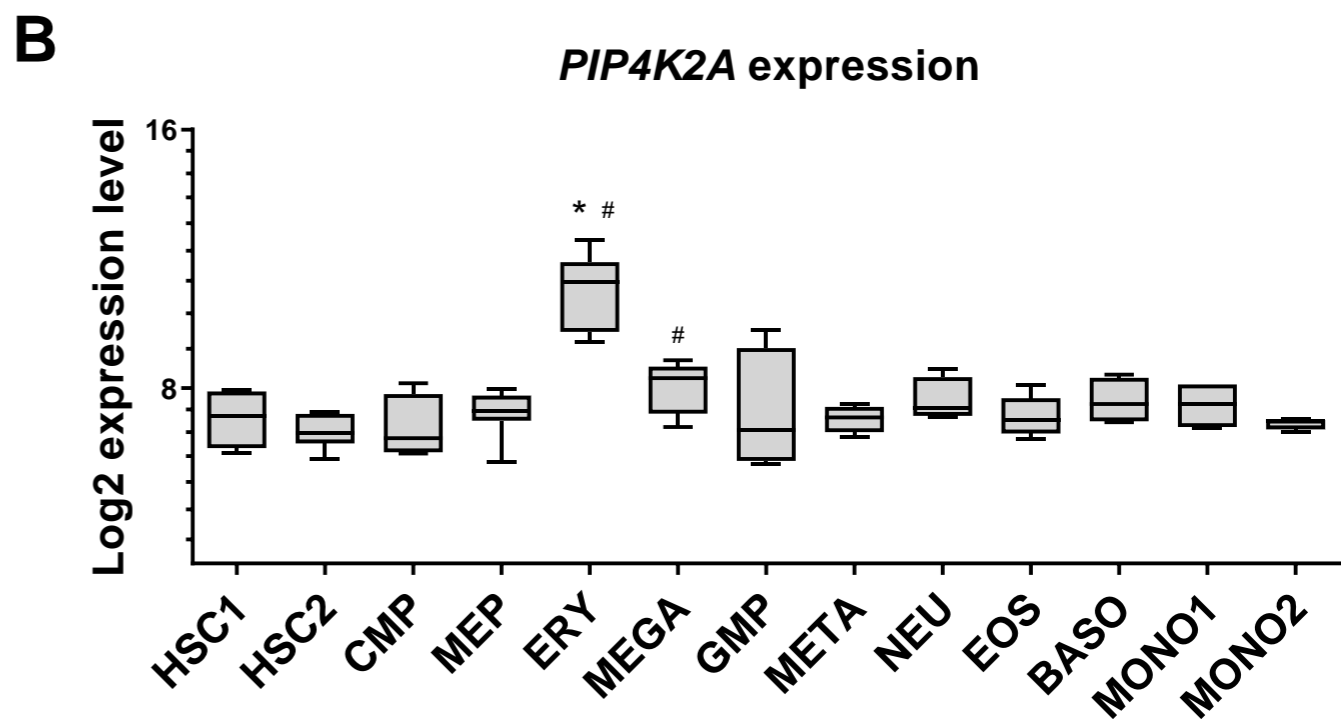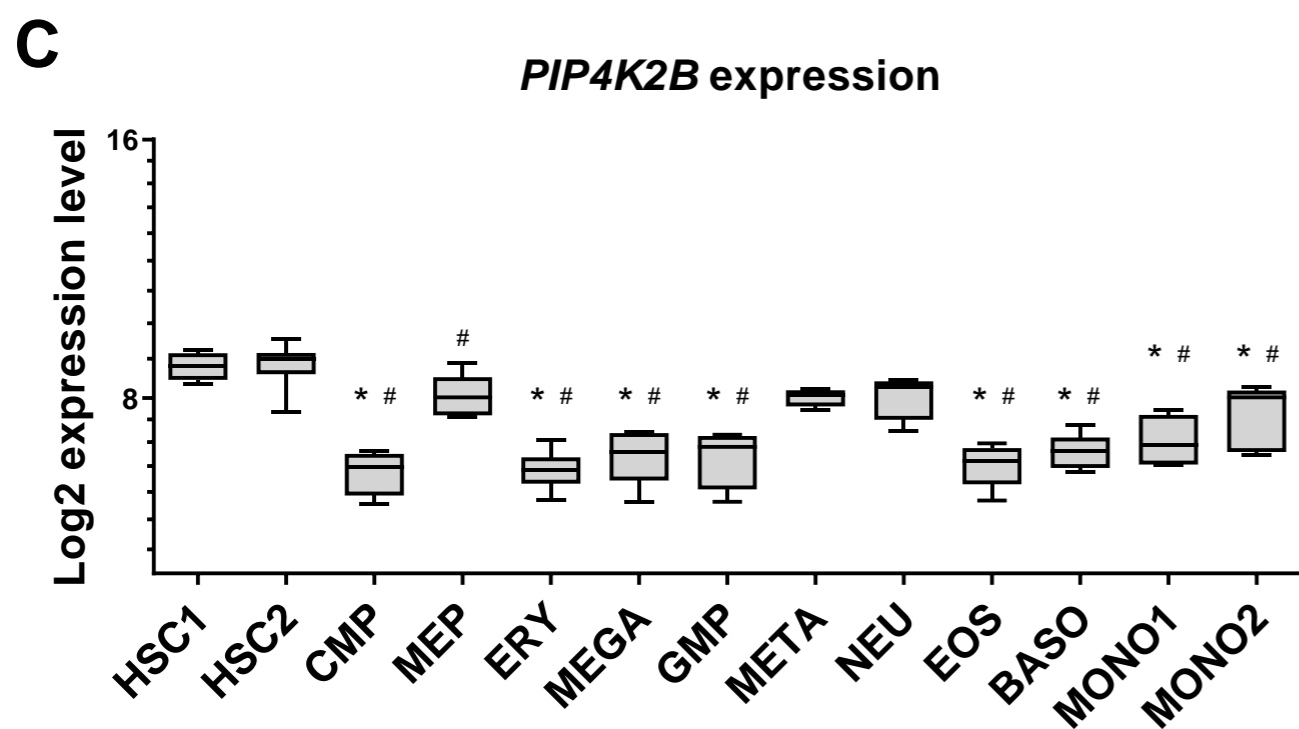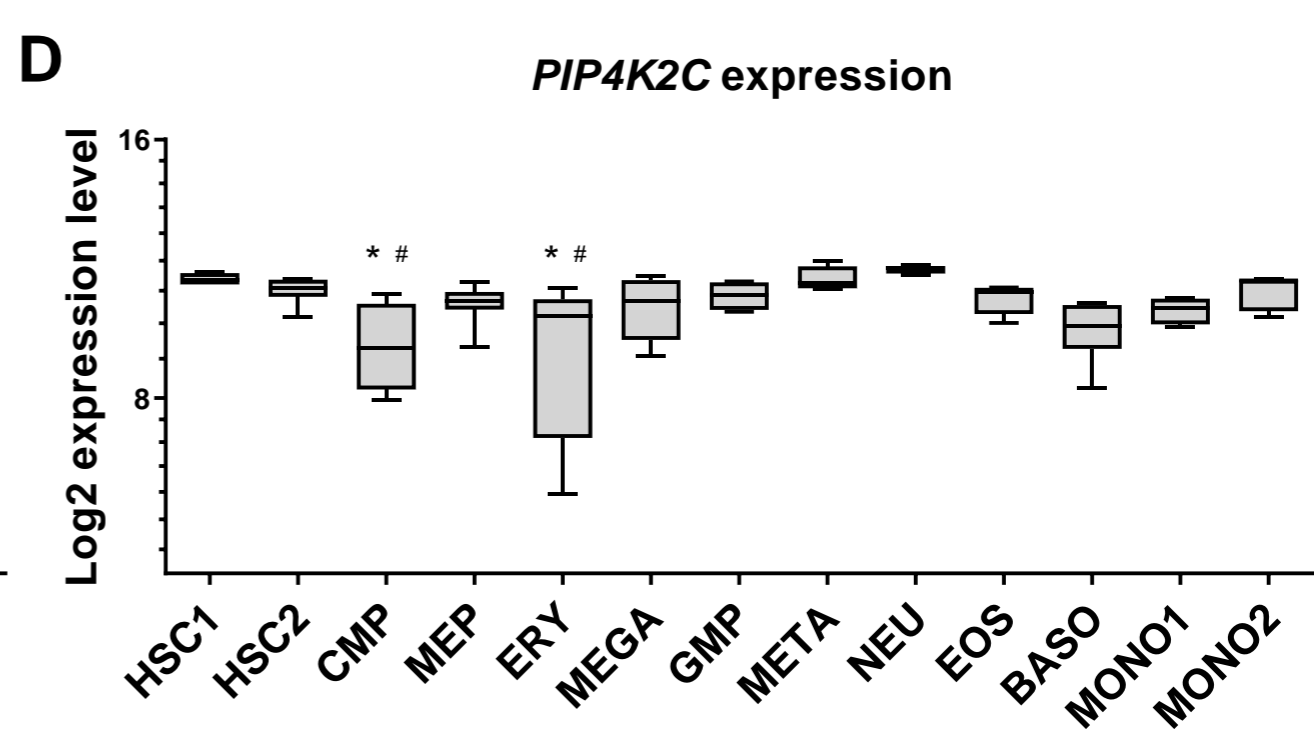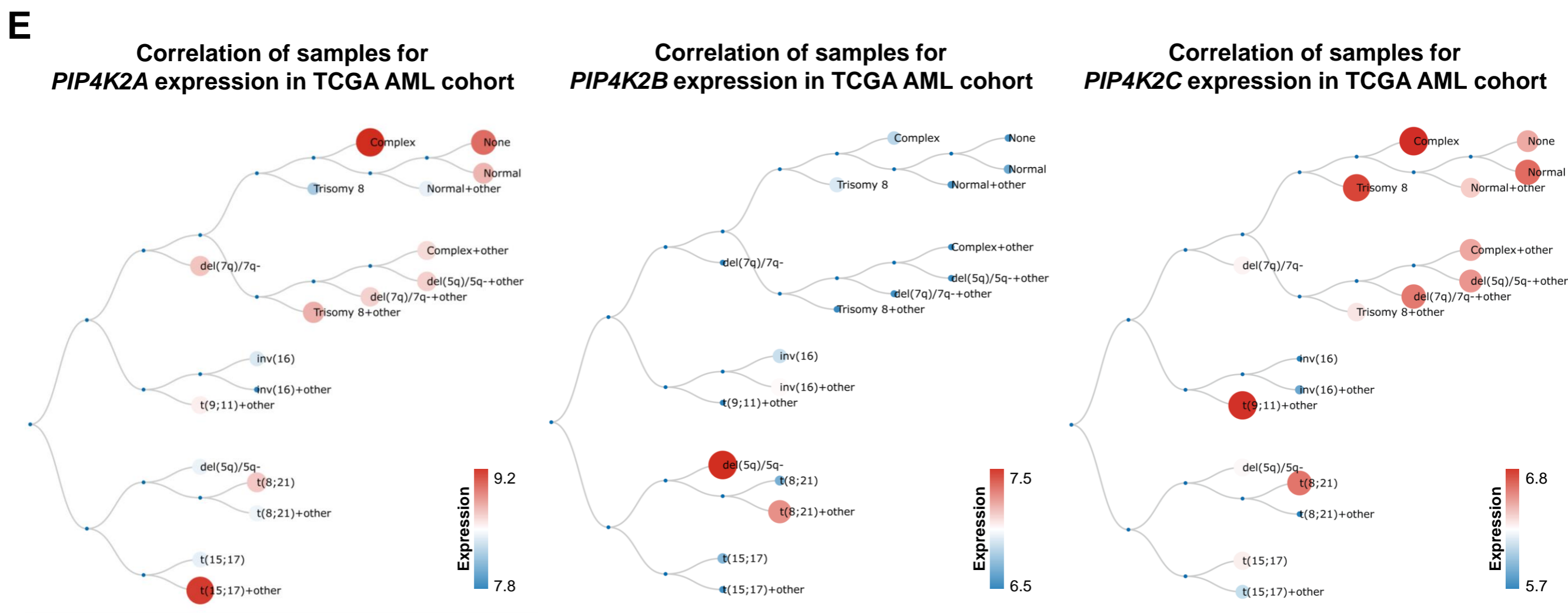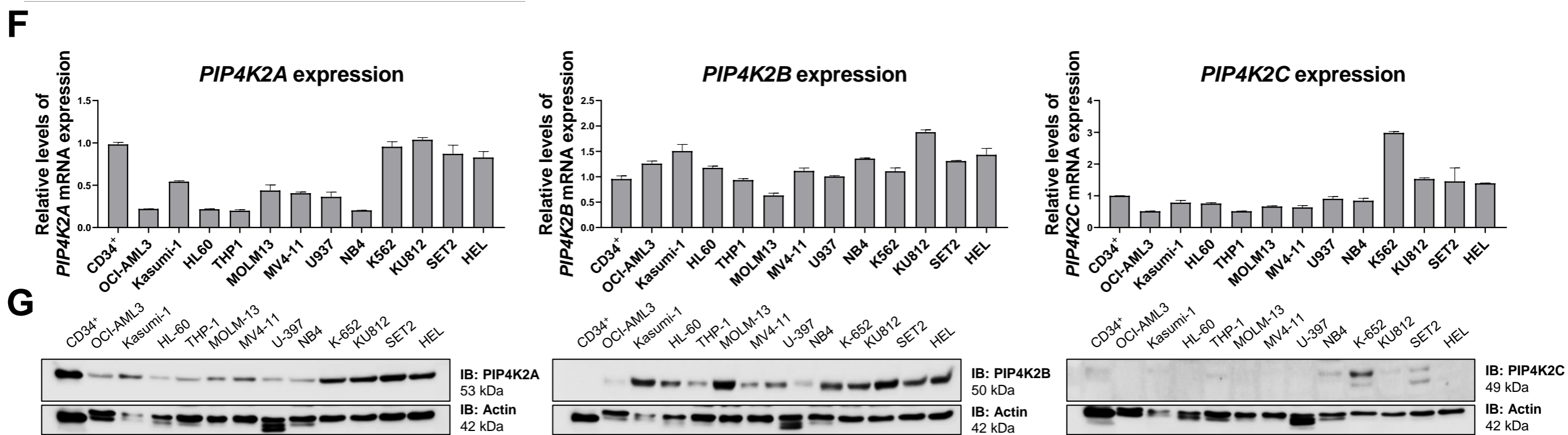

Supplement: Supplementary file 1 [file ijms-24-16899-s001.zip › Figure S1.pdf]

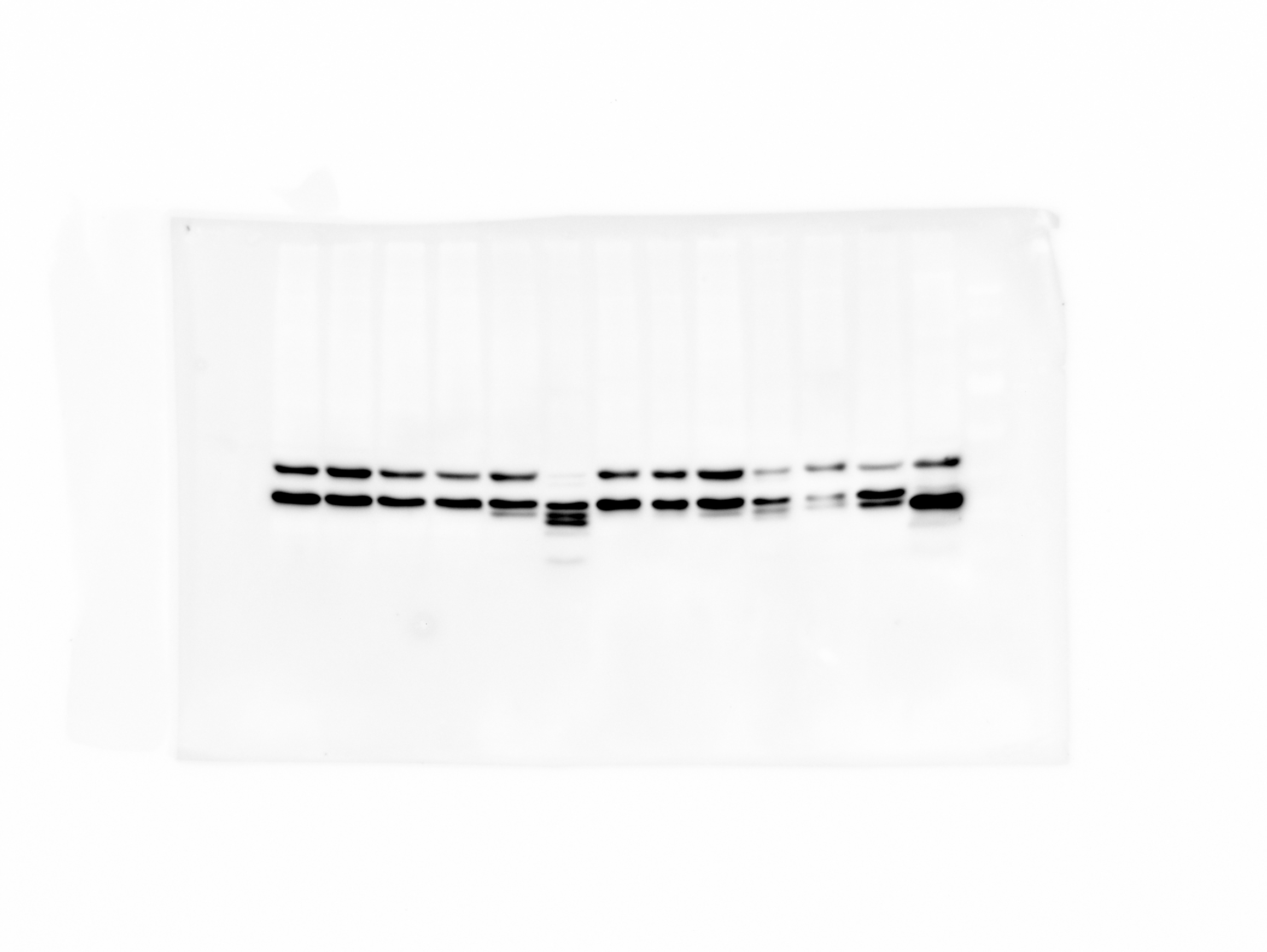

Supplement: Supplementary file 1 [file ijms-24-16899-s001.zip › WB_Whole Gels/Figure 1/Actin_PIP4K2A_LMA_Blot.tif]

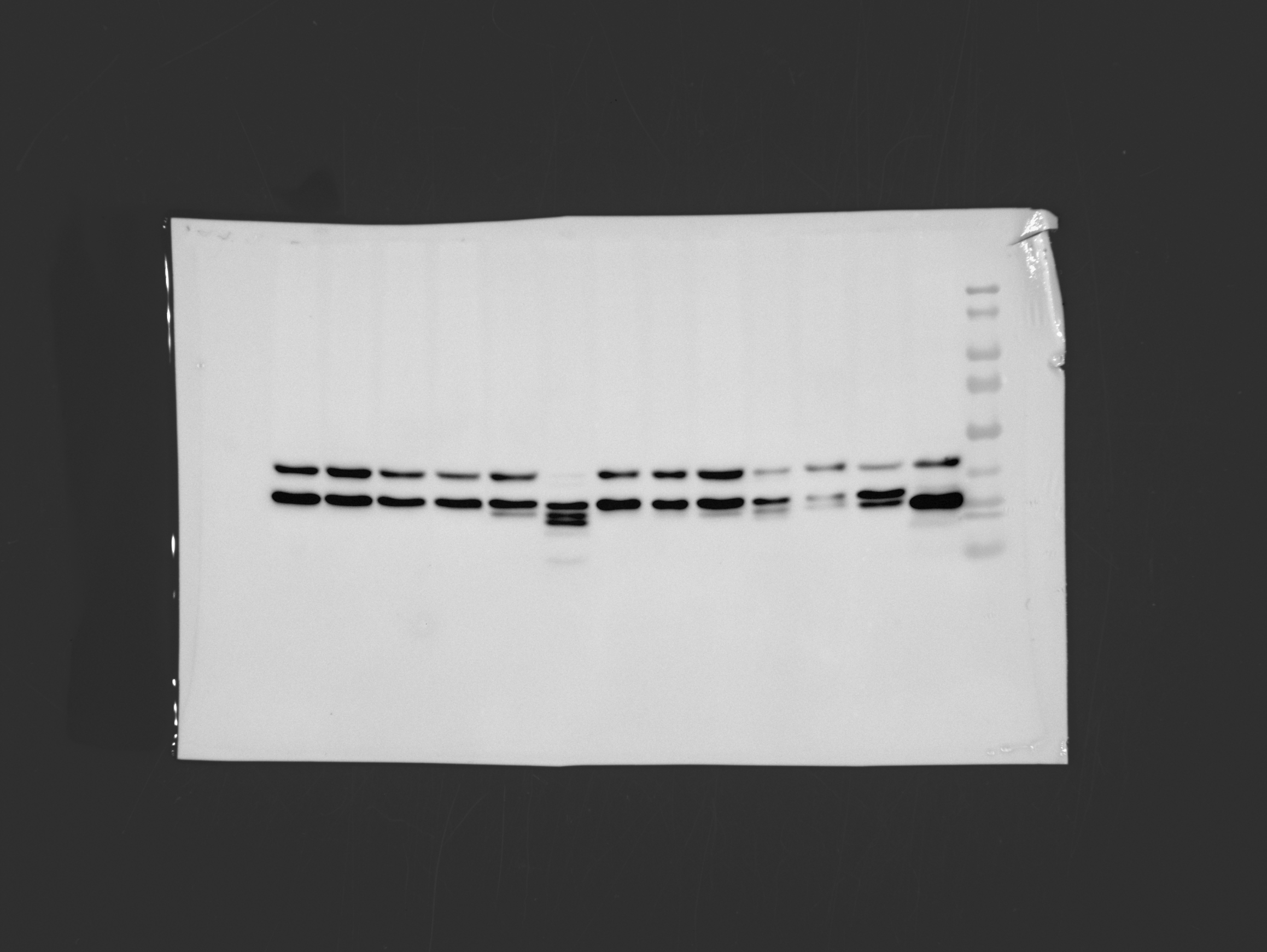

Supplement: Supplementary file 1 [file ijms-24-16899-s001.zip › WB_Whole Gels/Figure 1/Actin_PIP4K2A_LMA_Marker.tif]

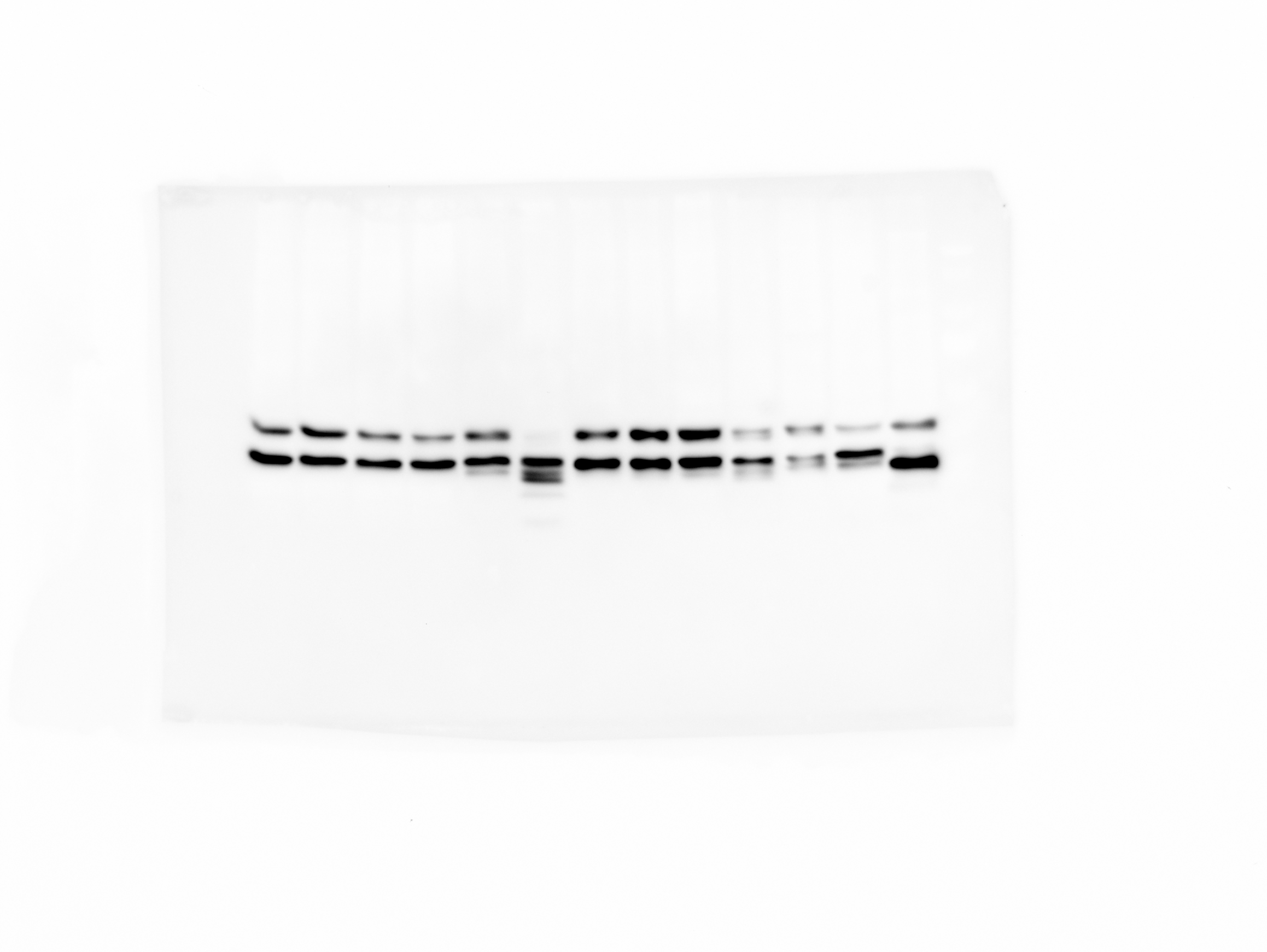

Supplement: Supplementary file 1 [file ijms-24-16899-s001.zip › WB_Whole Gels/Figure 1/Actin_PIP4K2B_LMA_Blot.tif]

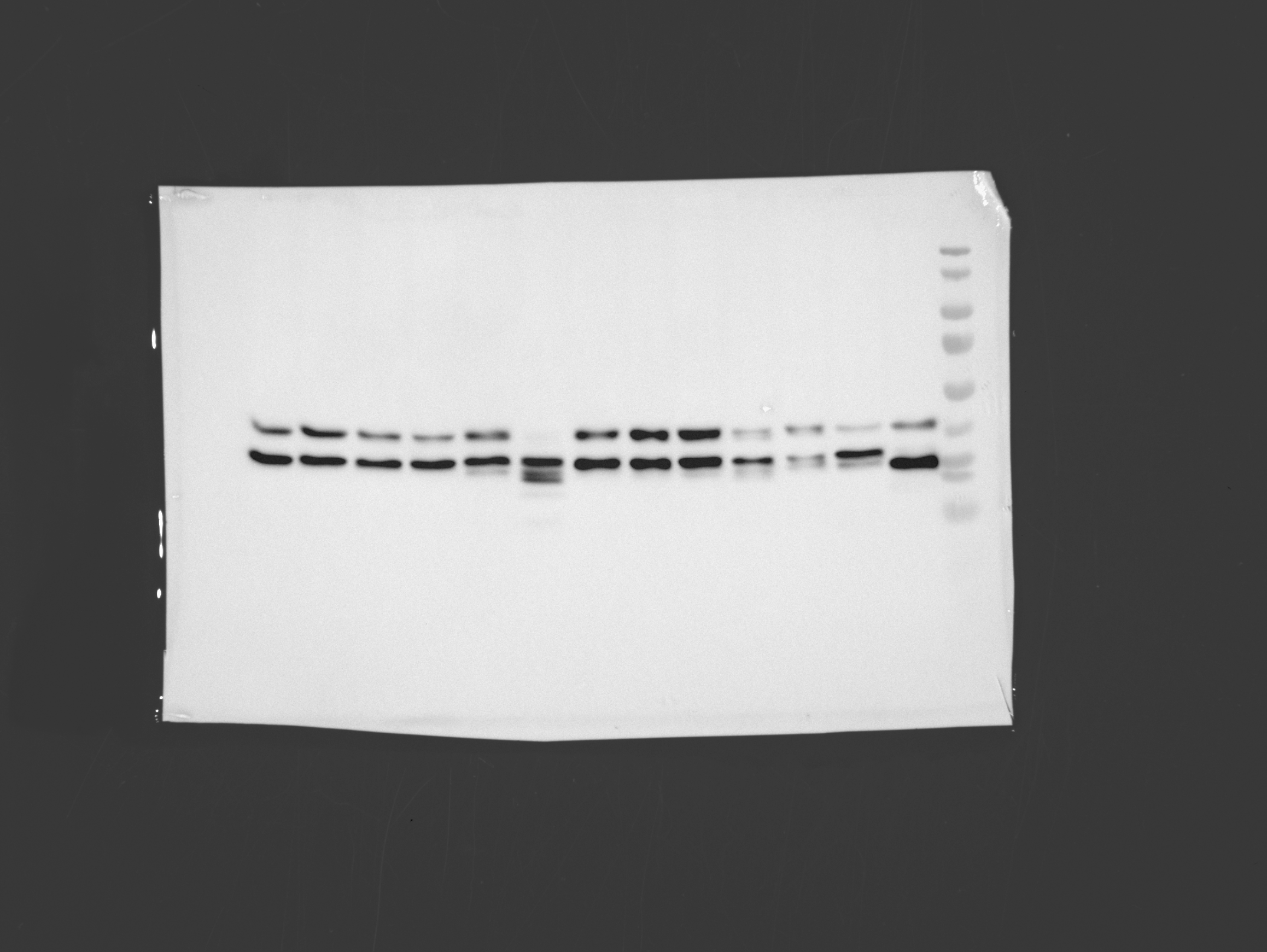

Supplement: Supplementary file 1 [file ijms-24-16899-s001.zip › WB_Whole Gels/Figure 1/Actin_PIP4K2B_LMA_Marker.tif]

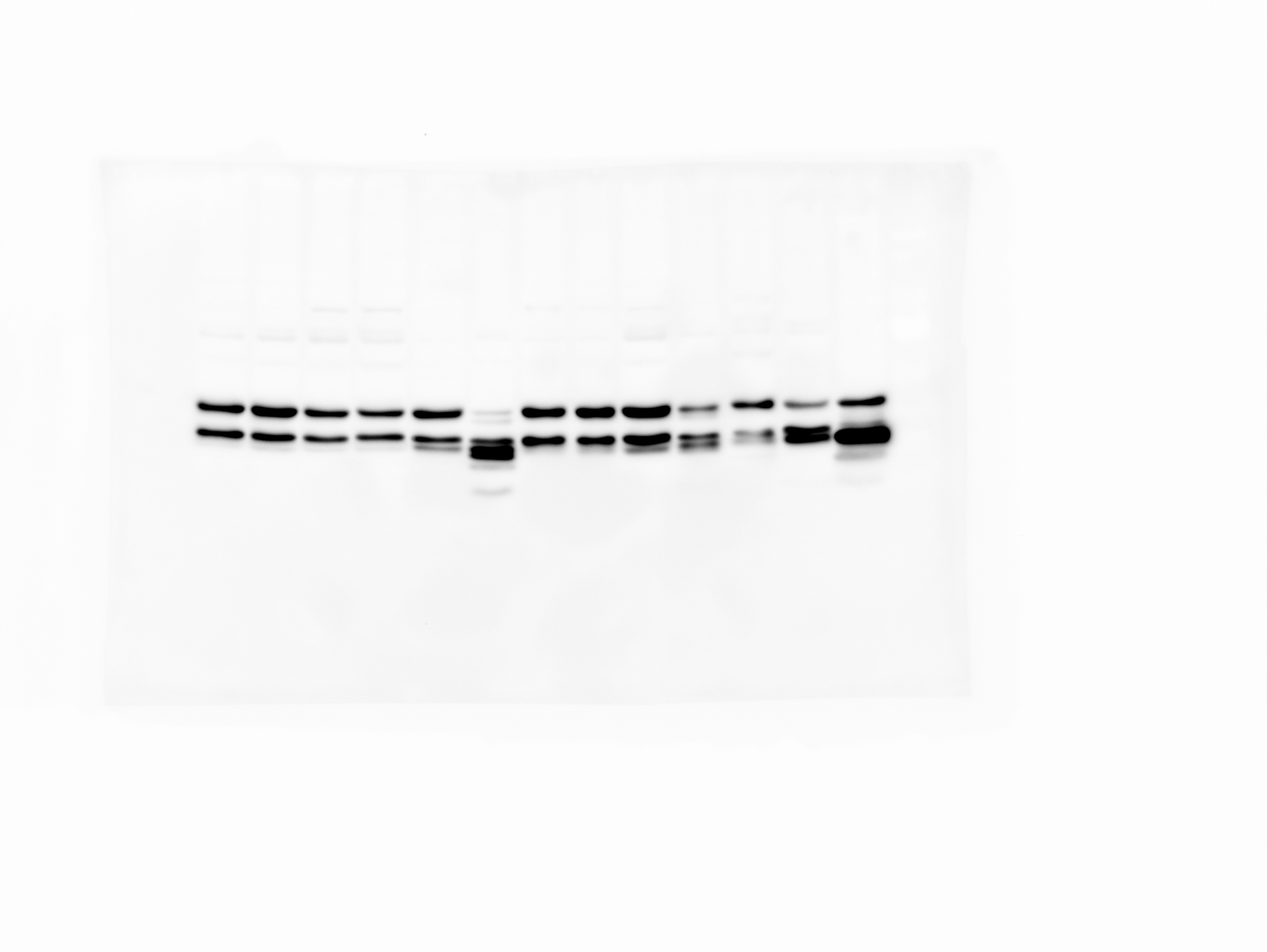

Supplement: Supplementary file 1 [file ijms-24-16899-s001.zip › WB_Whole Gels/Figure 1/Actin_PIP4K2C_LMA_Blot.tif]

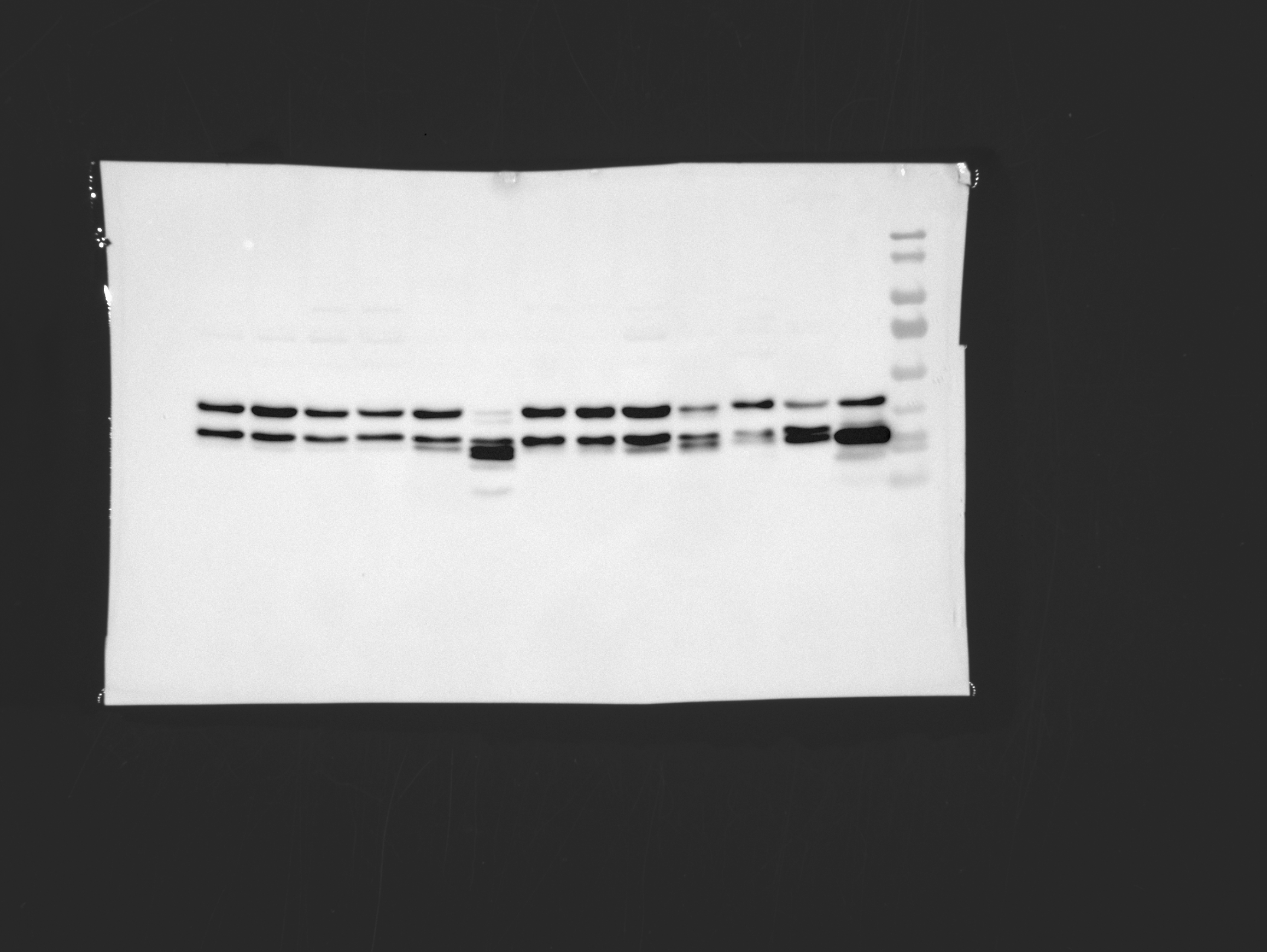

Supplement: Supplementary file 1 [file ijms-24-16899-s001.zip › WB_Whole Gels/Figure 1/Actin_PIP4K2C_LMA_Marker.tif]

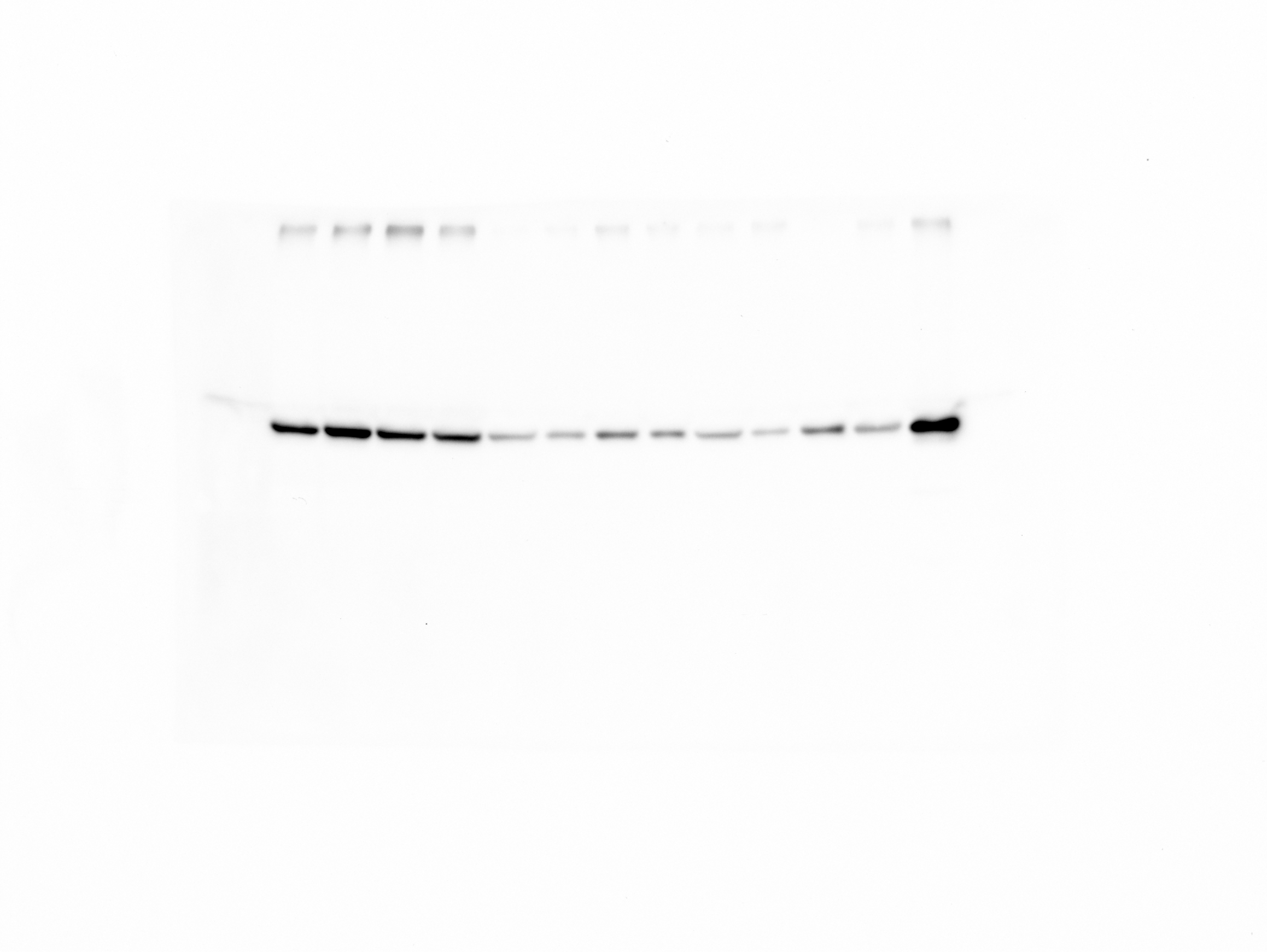

Supplement: Supplementary file 1 [file ijms-24-16899-s001.zip › WB_Whole Gels/Figure 1/PIP4K2A_LMA_Blot.tif]

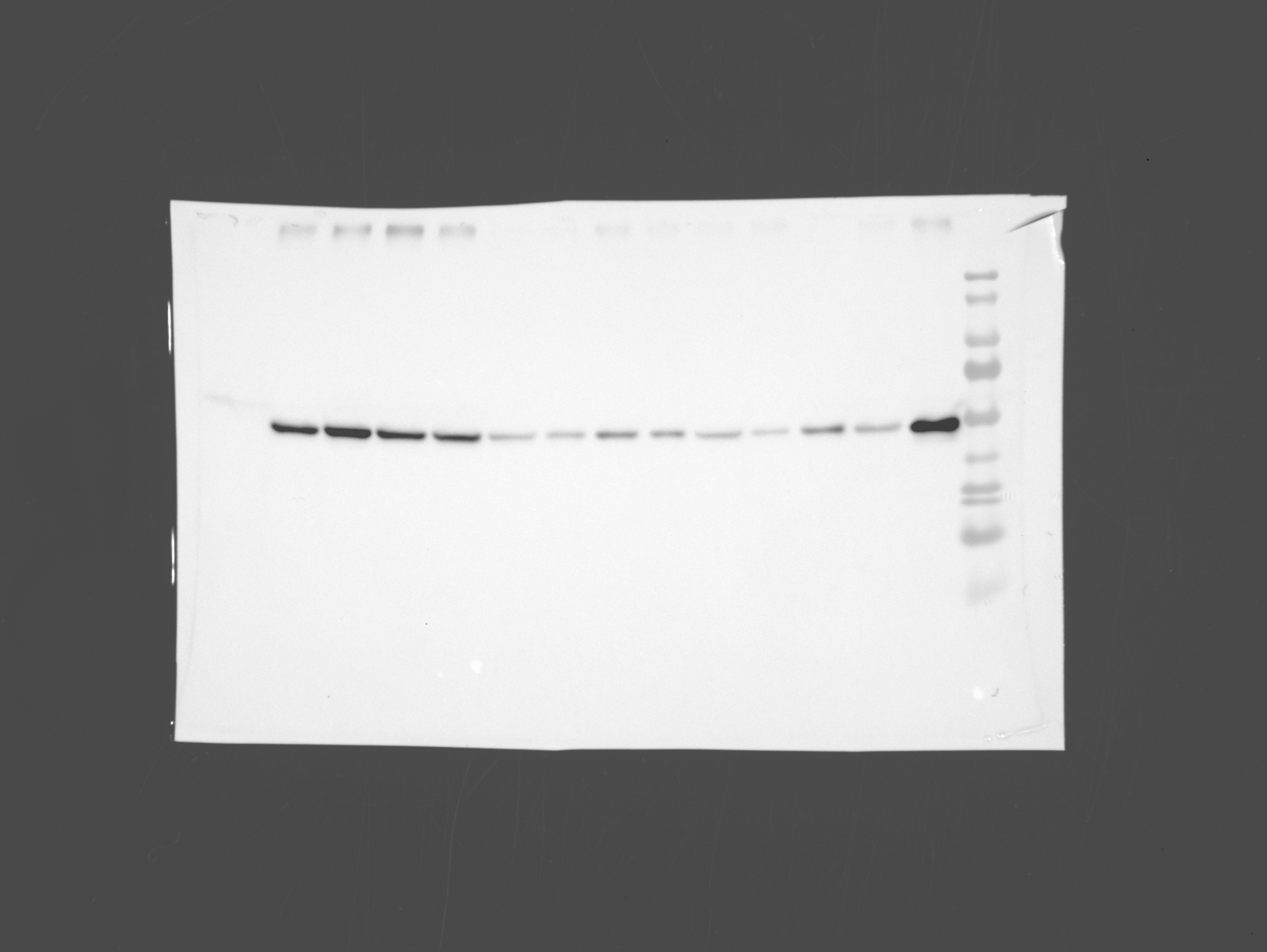

Supplement: Supplementary file 1 [file ijms-24-16899-s001.zip › WB_Whole Gels/Figure 1/PIP4K2A_LMA_Marker.tif]

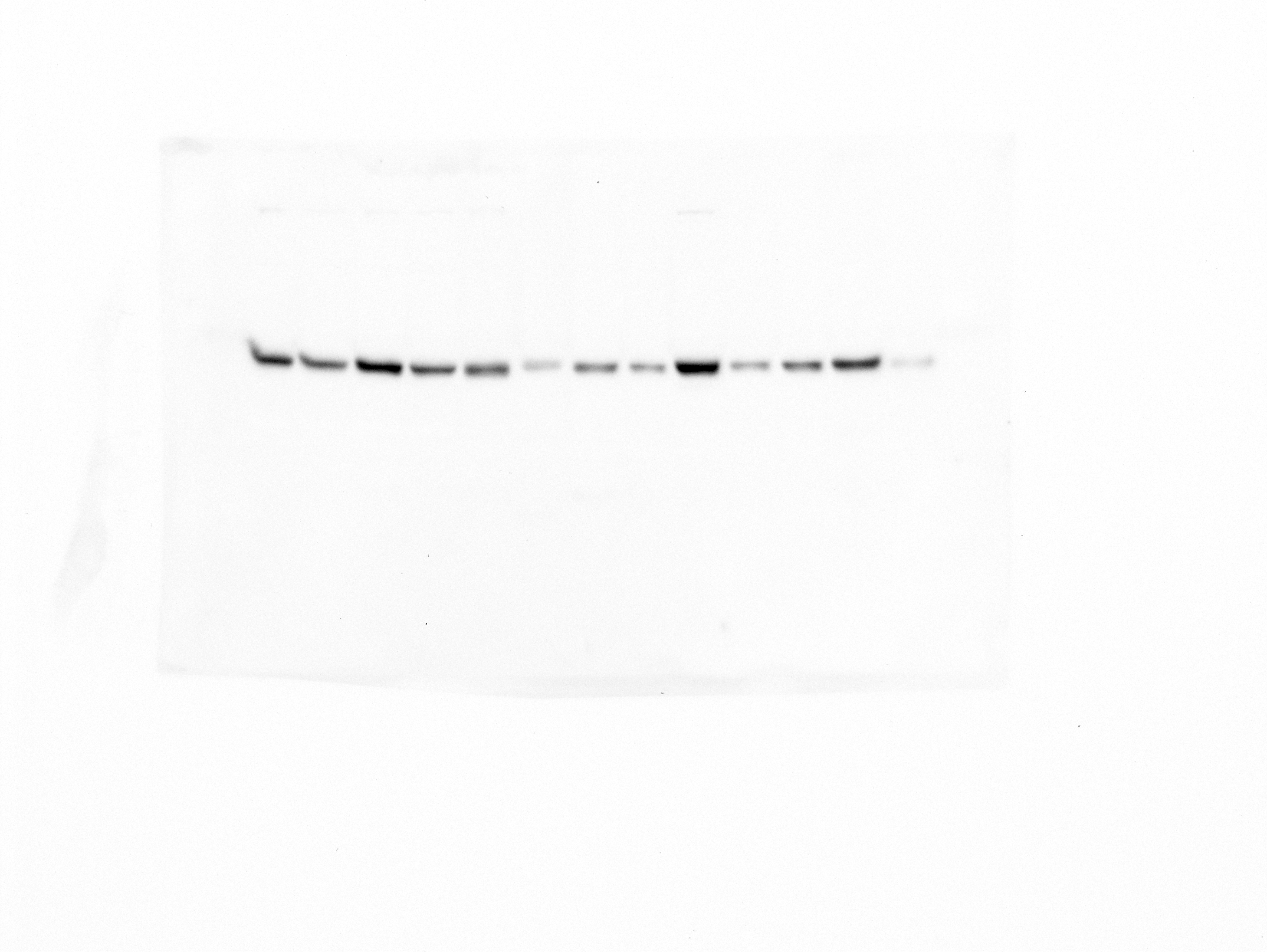

Supplement: Supplementary file 1 [file ijms-24-16899-s001.zip › WB_Whole Gels/Figure 1/PIP4K2B_LMA_Blot.tif]

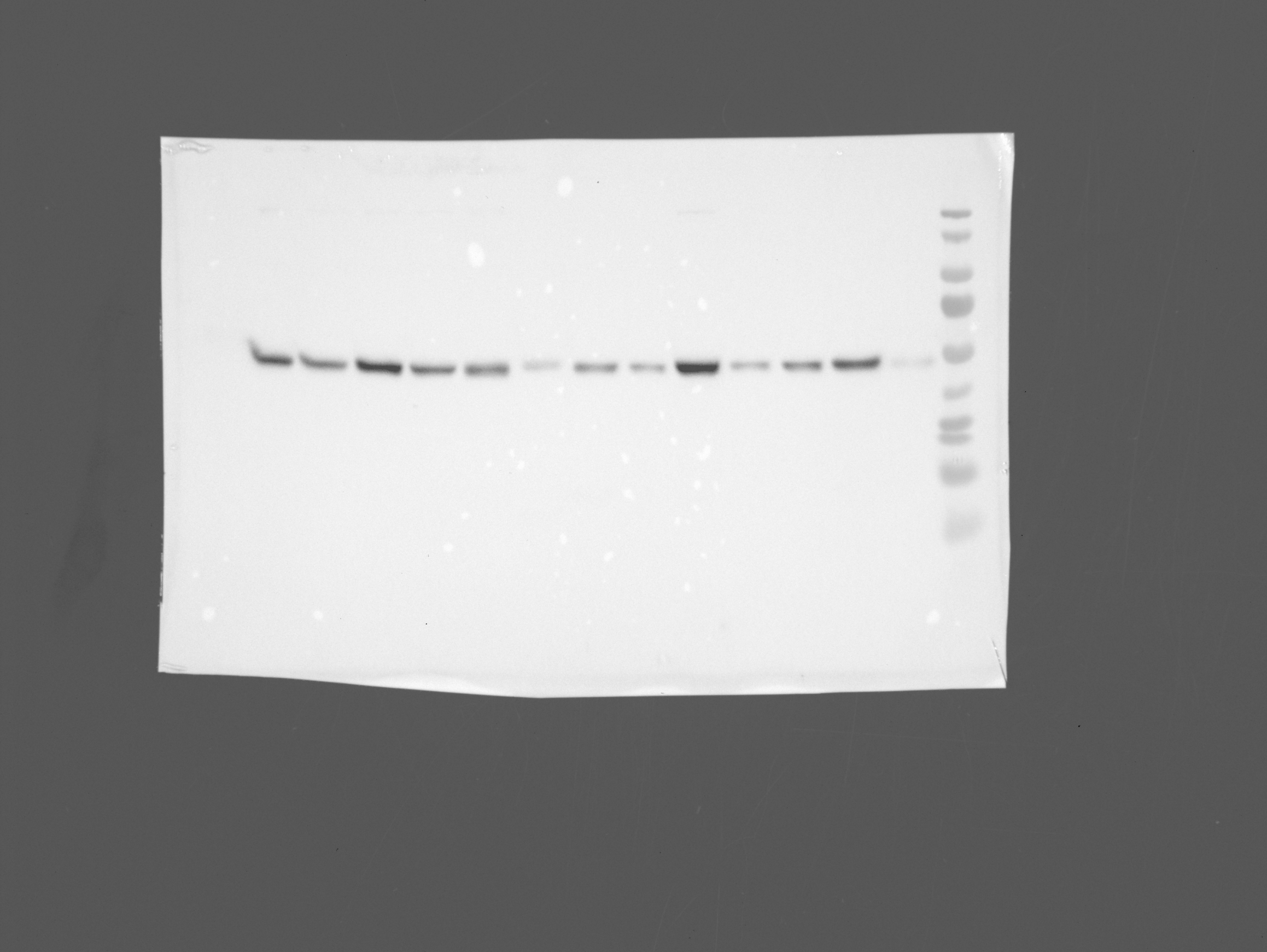

Supplement: Supplementary file 1 [file ijms-24-16899-s001.zip › WB_Whole Gels/Figure 1/PIP4K2B_LMA_Marker.tif]

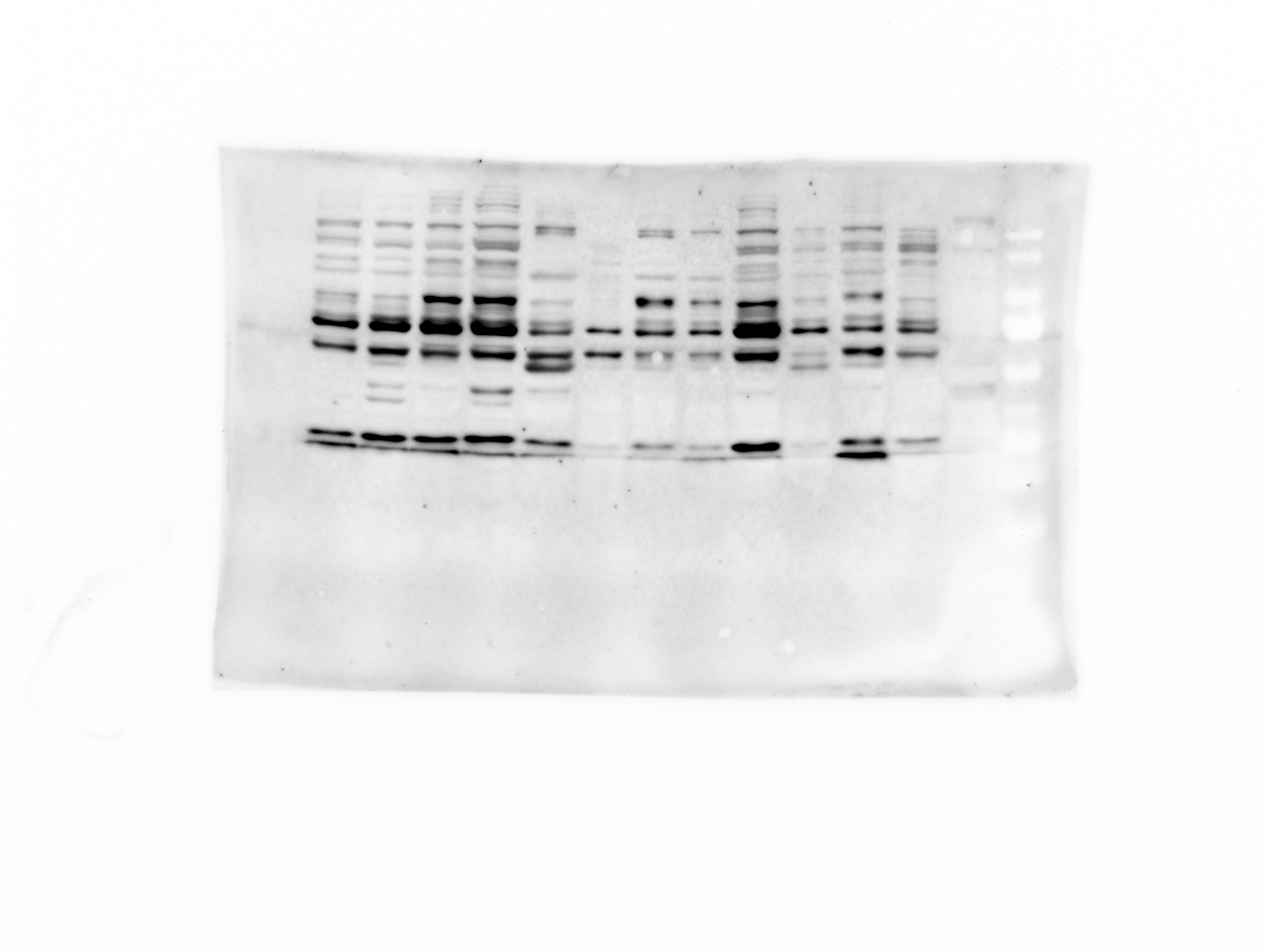

Supplement: Supplementary file 1 [file ijms-24-16899-s001.zip › WB_Whole Gels/Figure 1/PIP4K2C_LMA_Blot.tif]

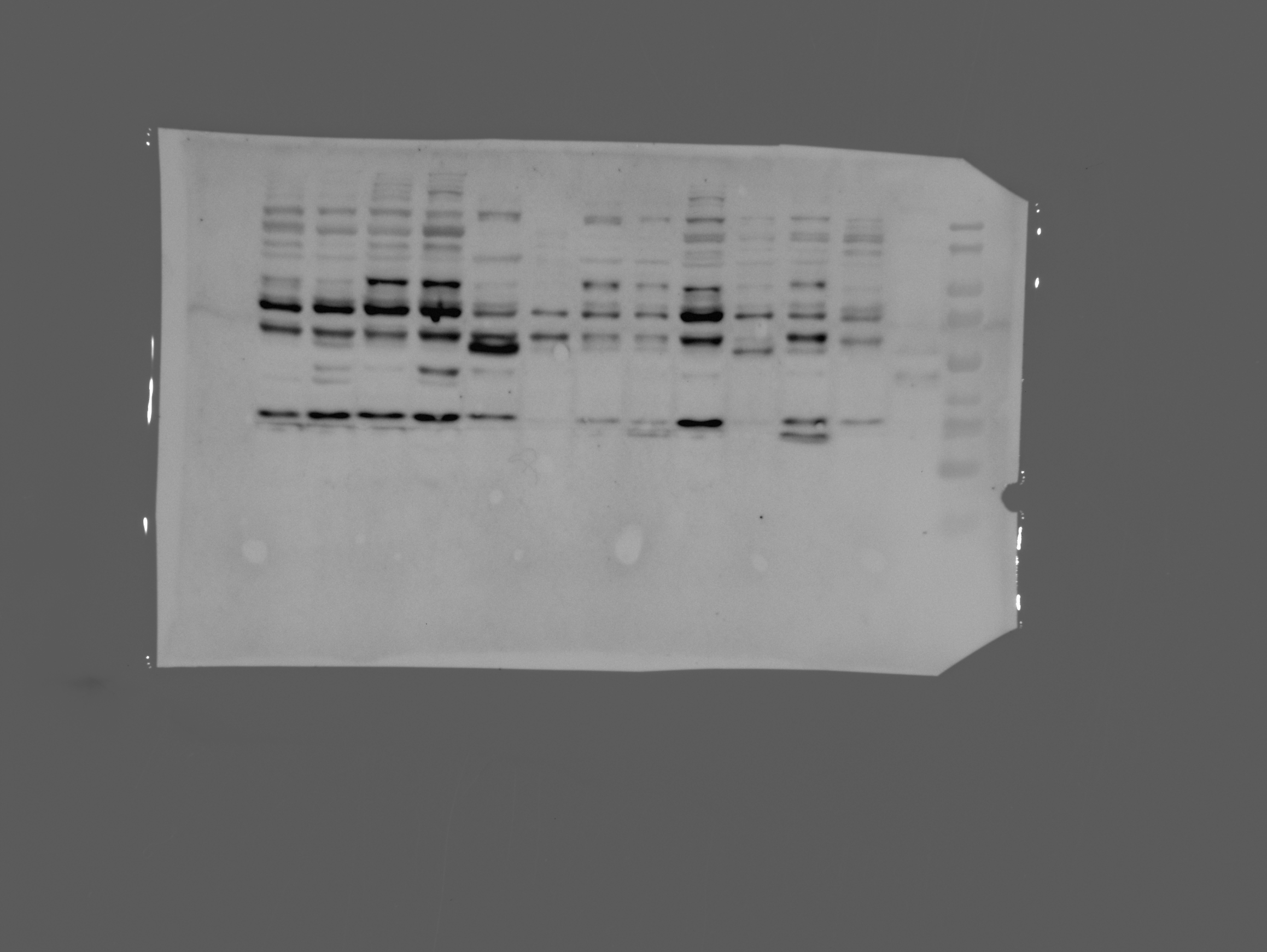

Supplement: Supplementary file 1 [file ijms-24-16899-s001.zip › WB_Whole Gels/Figure 1/PIP4K2C_LMA_Gel_2_Marker.tif]

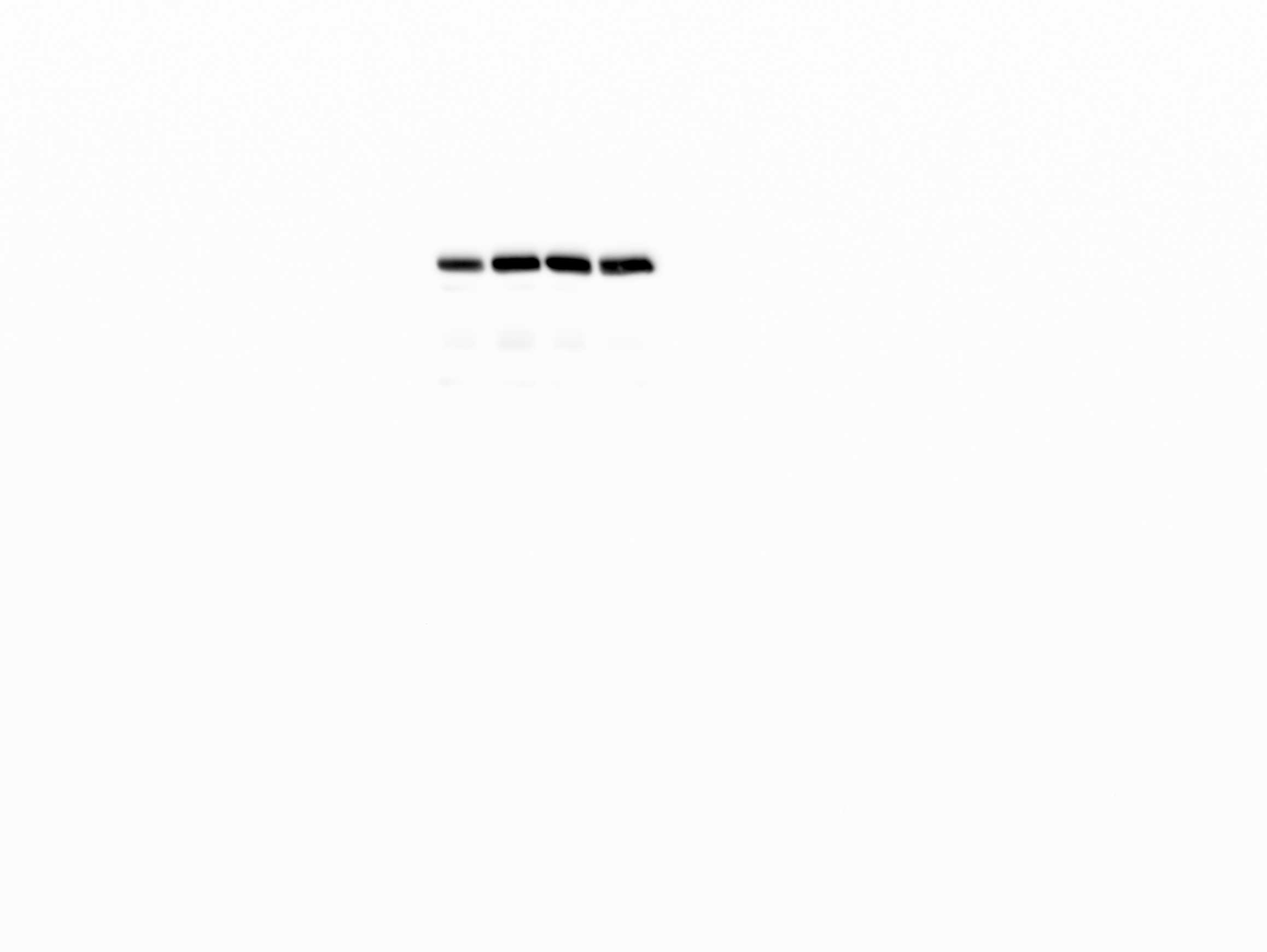

Supplement: Supplementary file 1 [file ijms-24-16899-s001.zip › WB_Whole Gels/Figure 3/a_Tubulina_KS1_10%_Blot.tif]

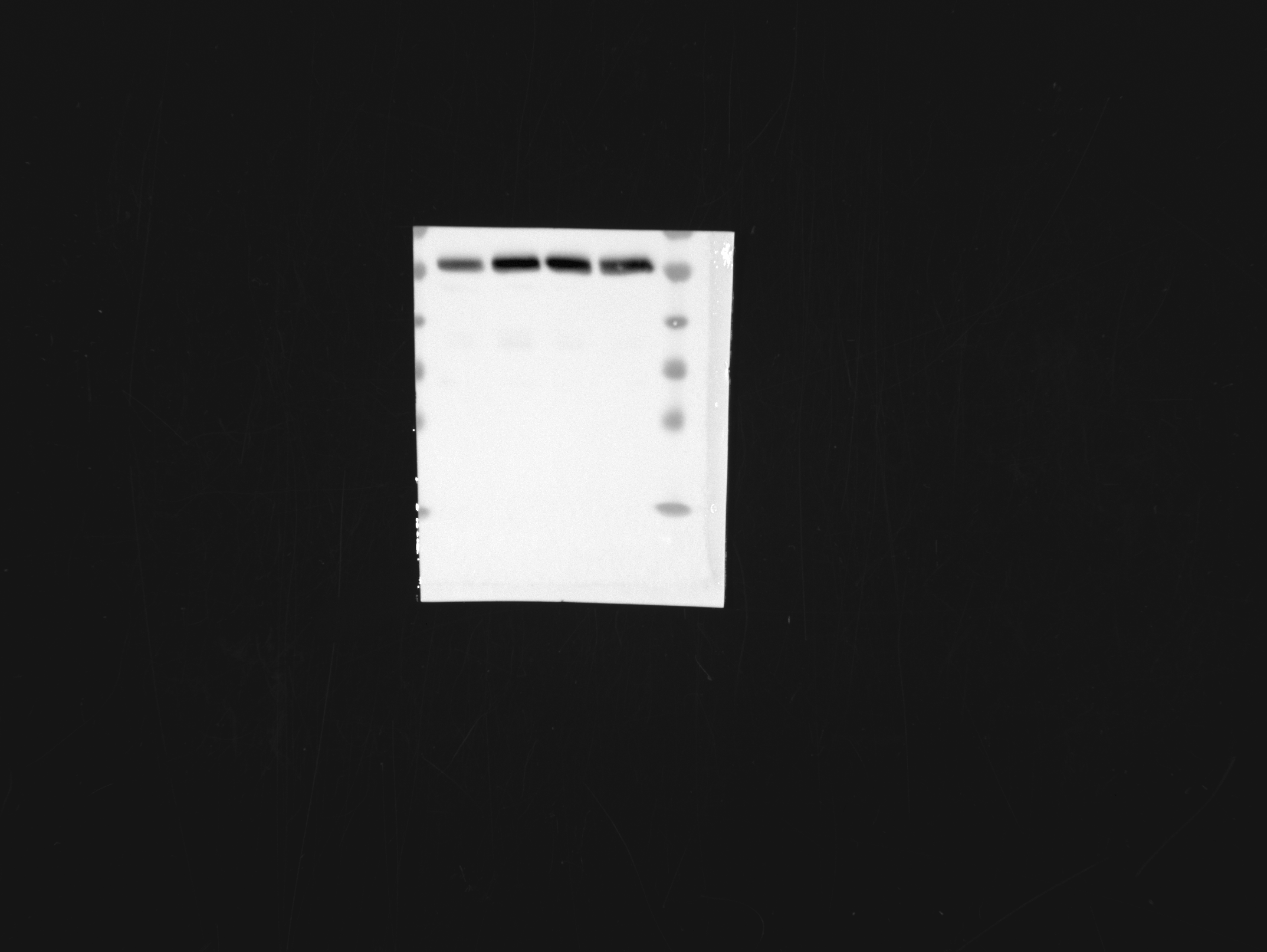

Supplement: Supplementary file 1 [file ijms-24-16899-s001.zip › WB_Whole Gels/Figure 3/a_Tubulina_KS1_10%_Marker.tif]

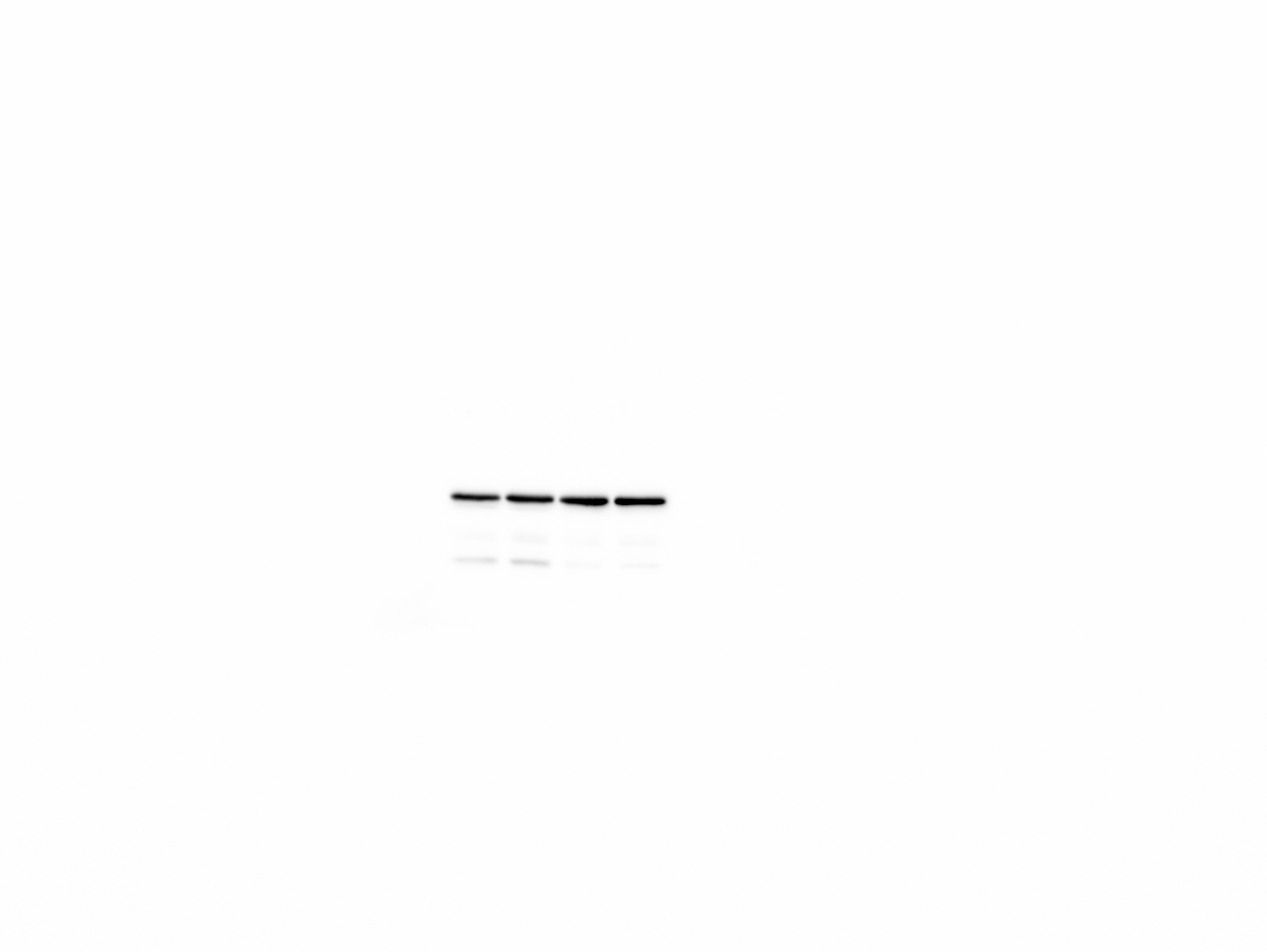

Supplement: Supplementary file 1 [file ijms-24-16899-s001.zip › WB_Whole Gels/Figure 3/a_Tubulina_KS1_15%_Blot.tif]

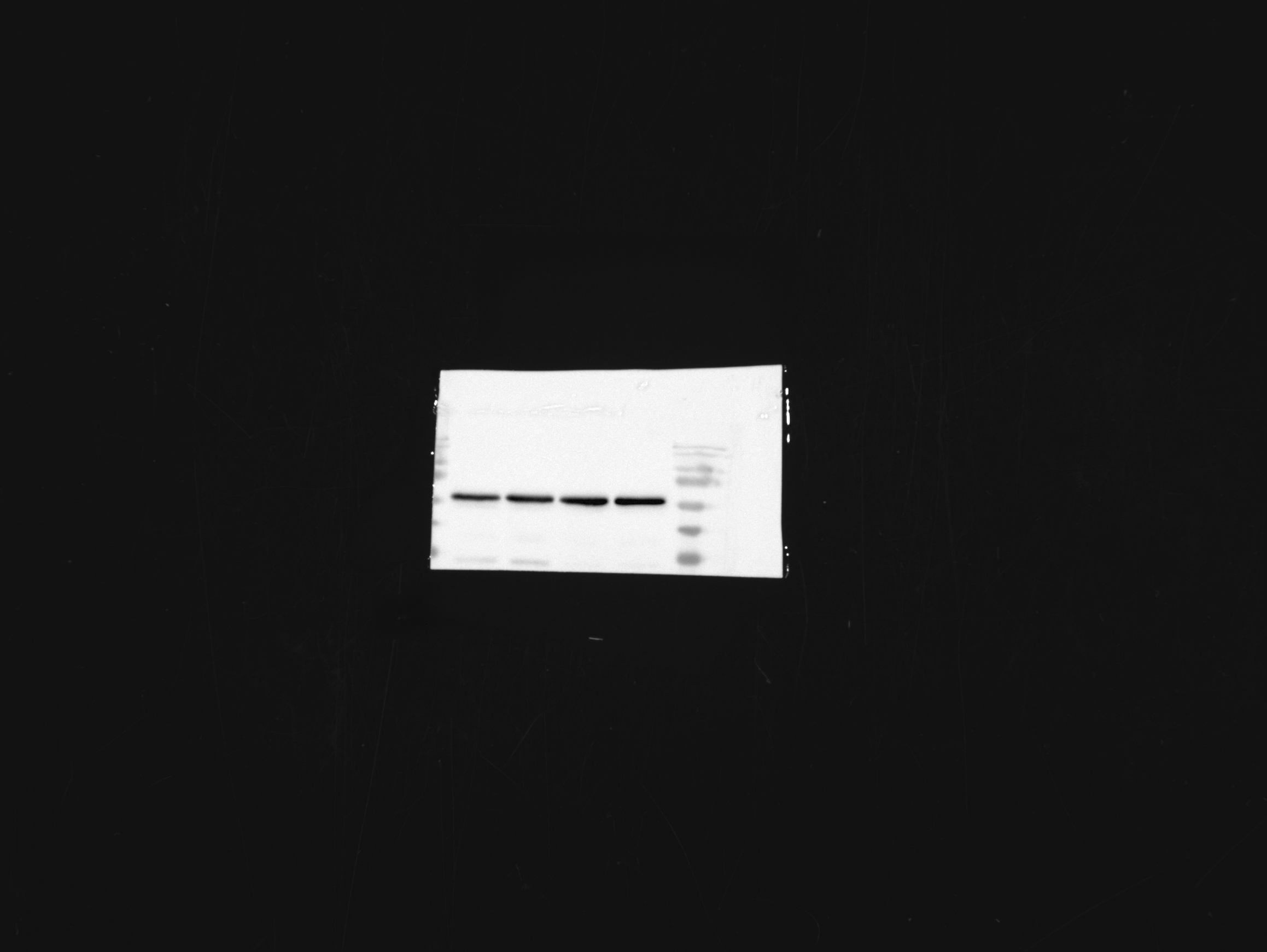

Supplement: Supplementary file 1 [file ijms-24-16899-s001.zip › WB_Whole Gels/Figure 3/a_Tubulina_KS1_15%_Marker.tif]

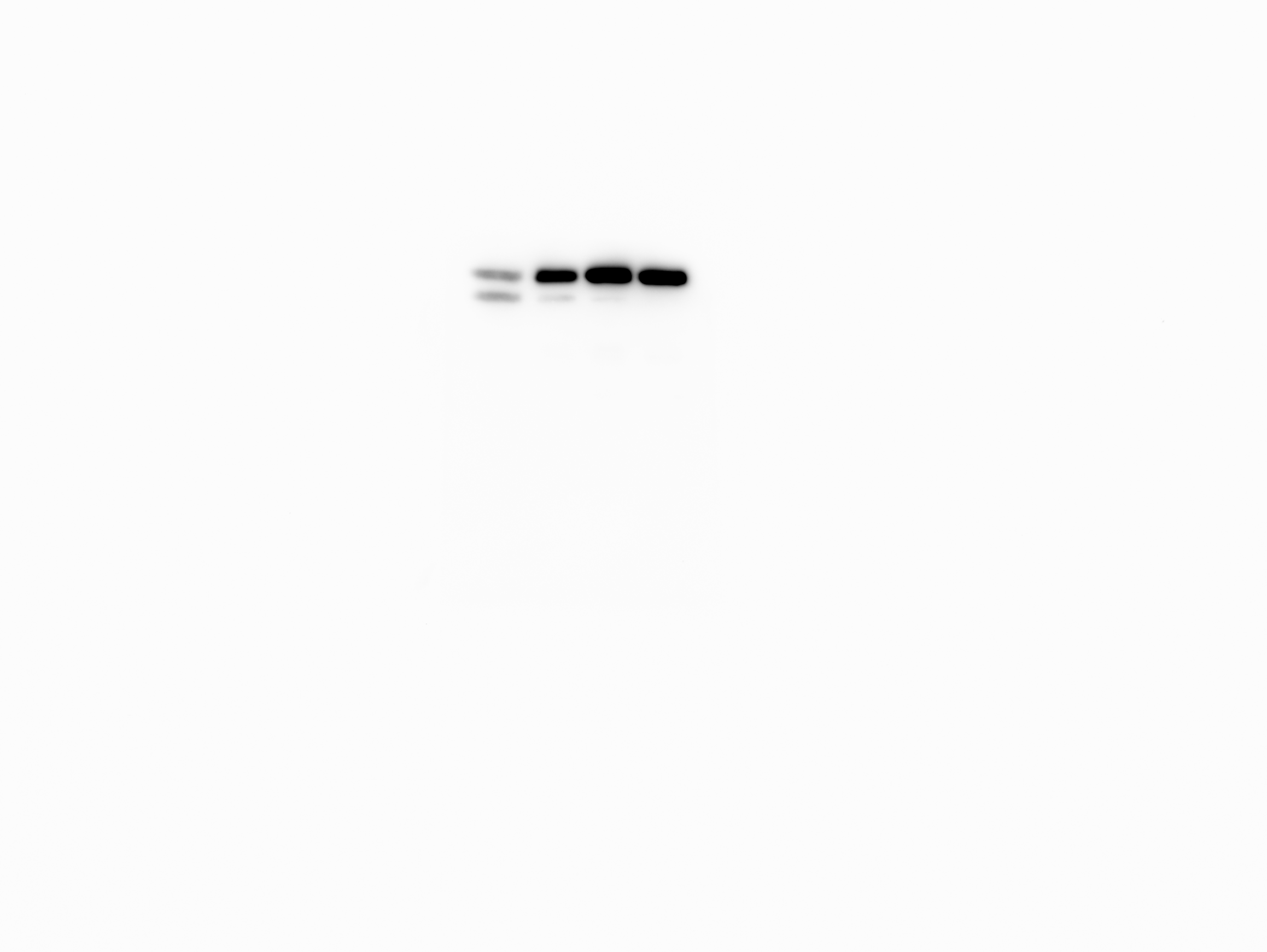

Supplement: Supplementary file 1 [file ijms-24-16899-s001.zip › WB_Whole Gels/Figure 3/a_Tubulina_NB4_10%_Blot.tif]

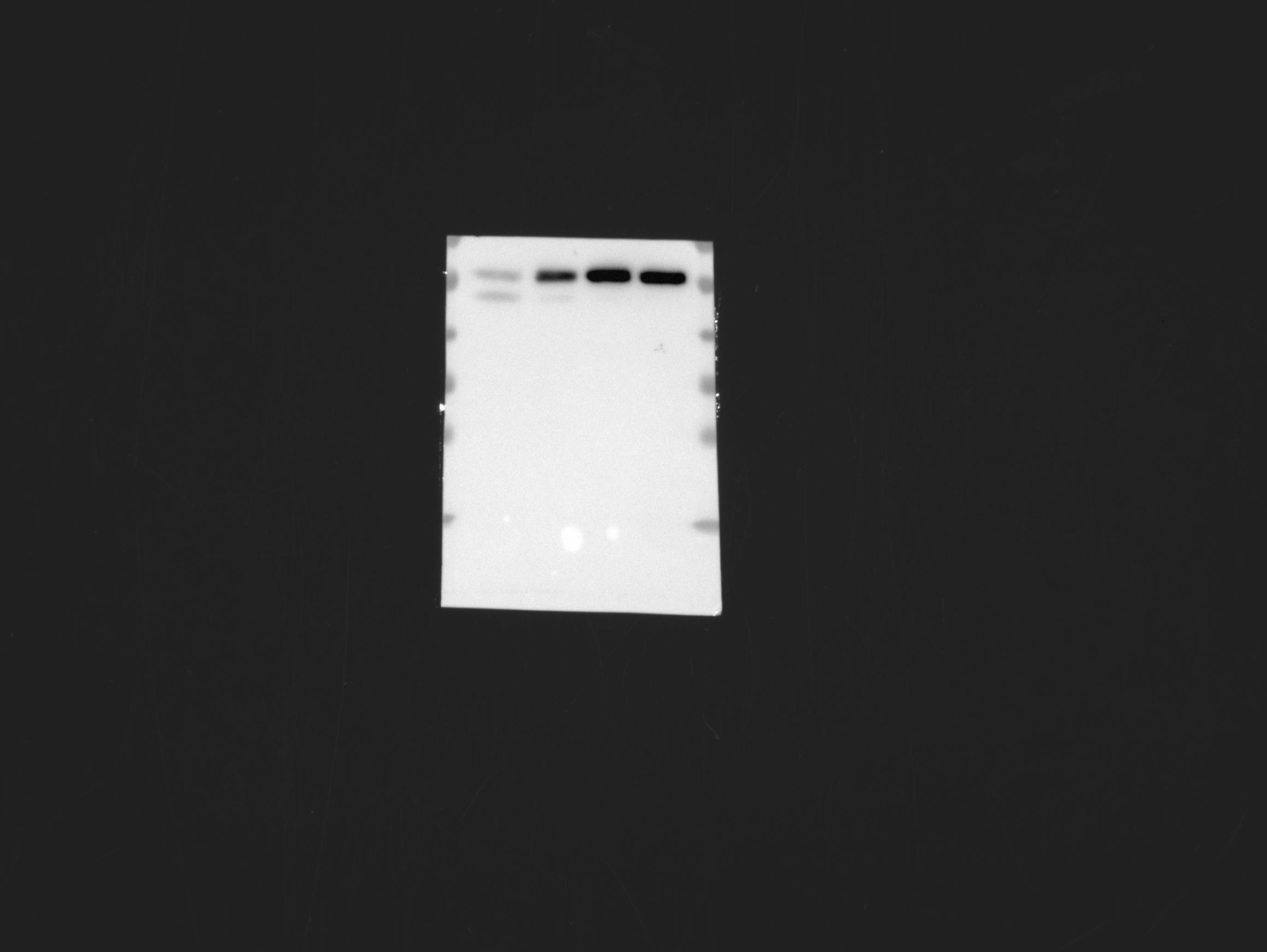

Supplement: Supplementary file 1 [file ijms-24-16899-s001.zip › WB_Whole Gels/Figure 3/a_Tubulina_NB4_10%_Marker.tif]

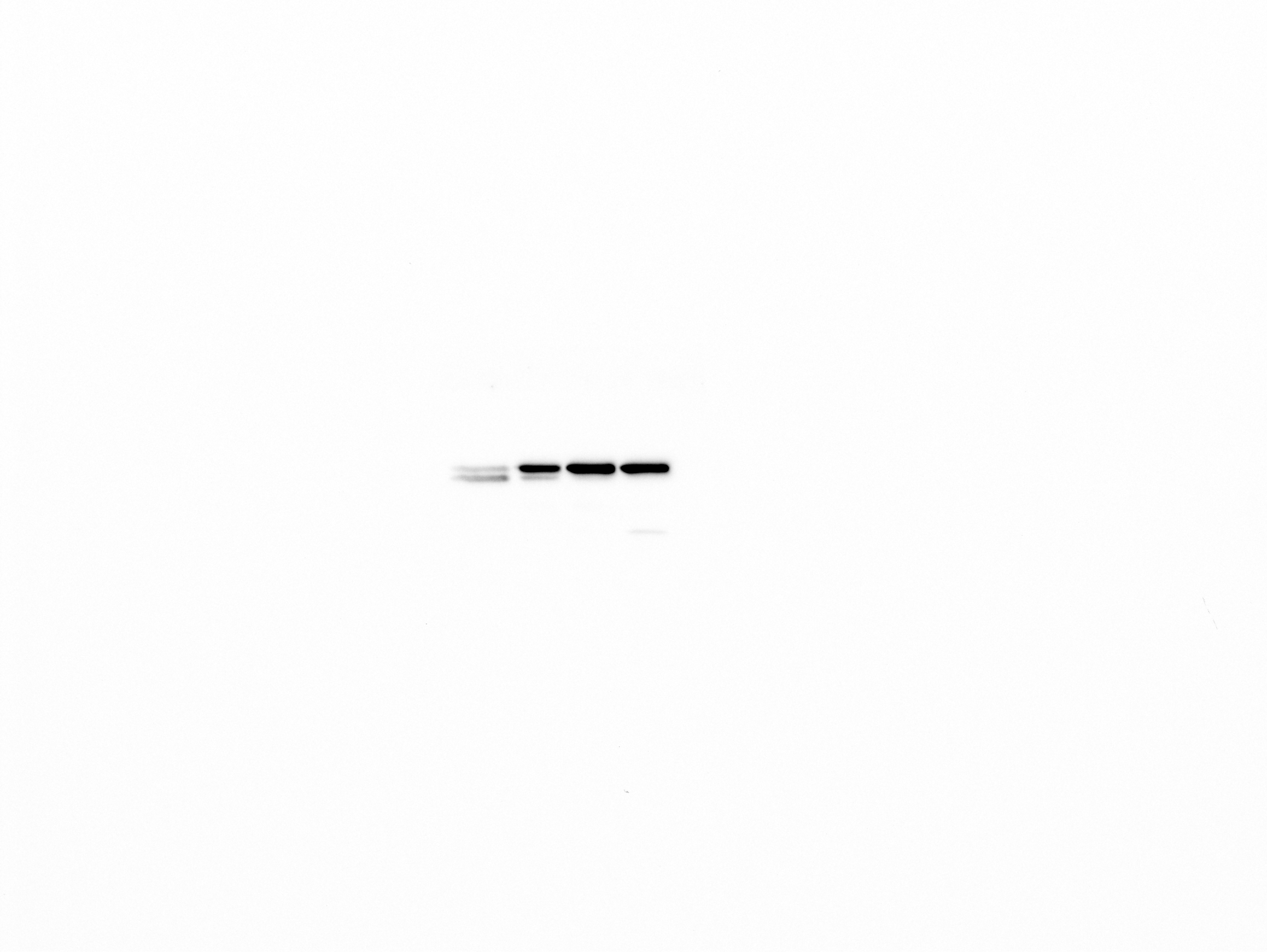

Supplement: Supplementary file 1 [file ijms-24-16899-s001.zip › WB_Whole Gels/Figure 3/a_Tubulina_NB4_15%_Blot.tif]

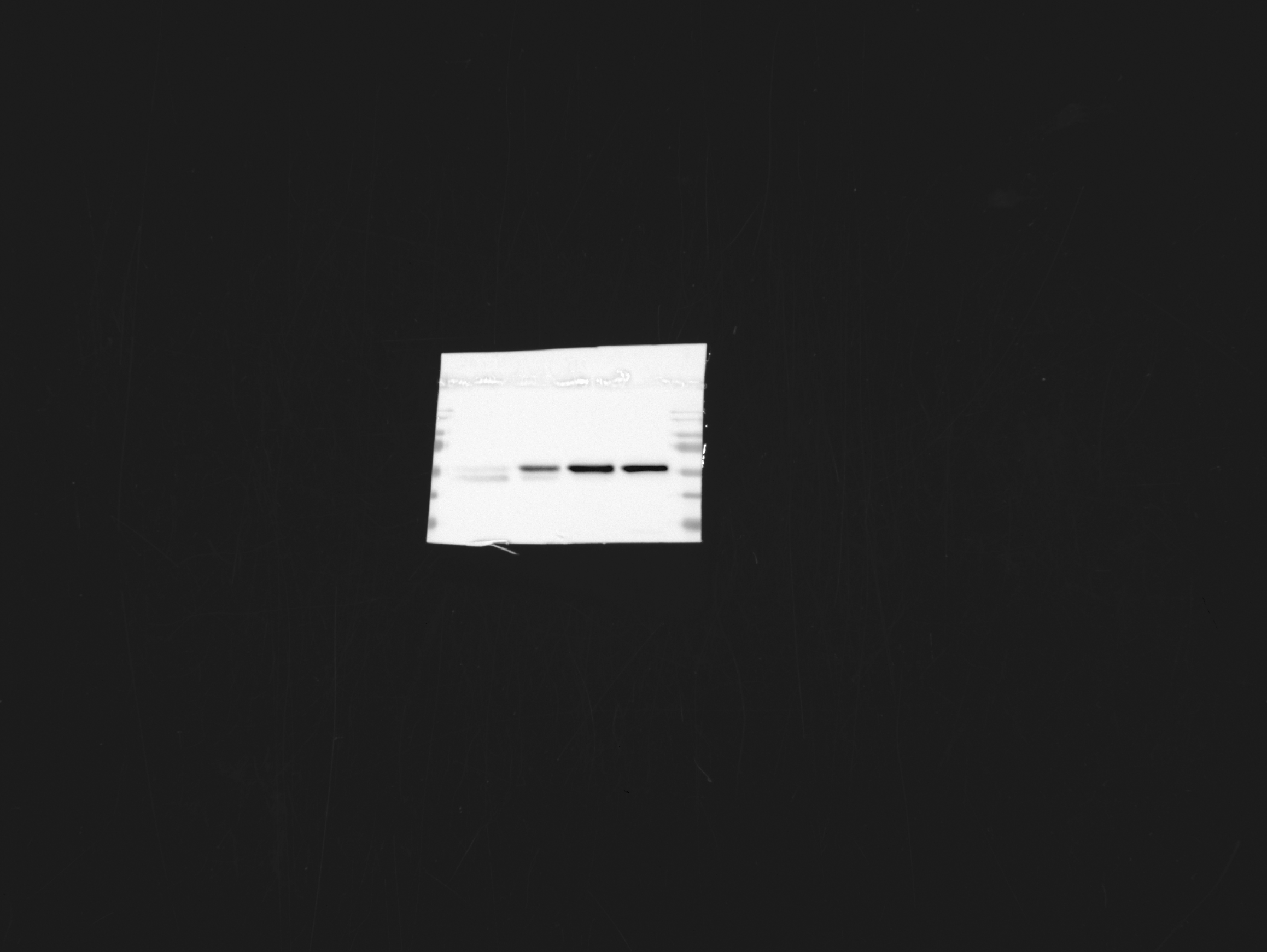

Supplement: Supplementary file 1 [file ijms-24-16899-s001.zip › WB_Whole Gels/Figure 3/a_Tubulina_NB4_15%_Marker.tif]

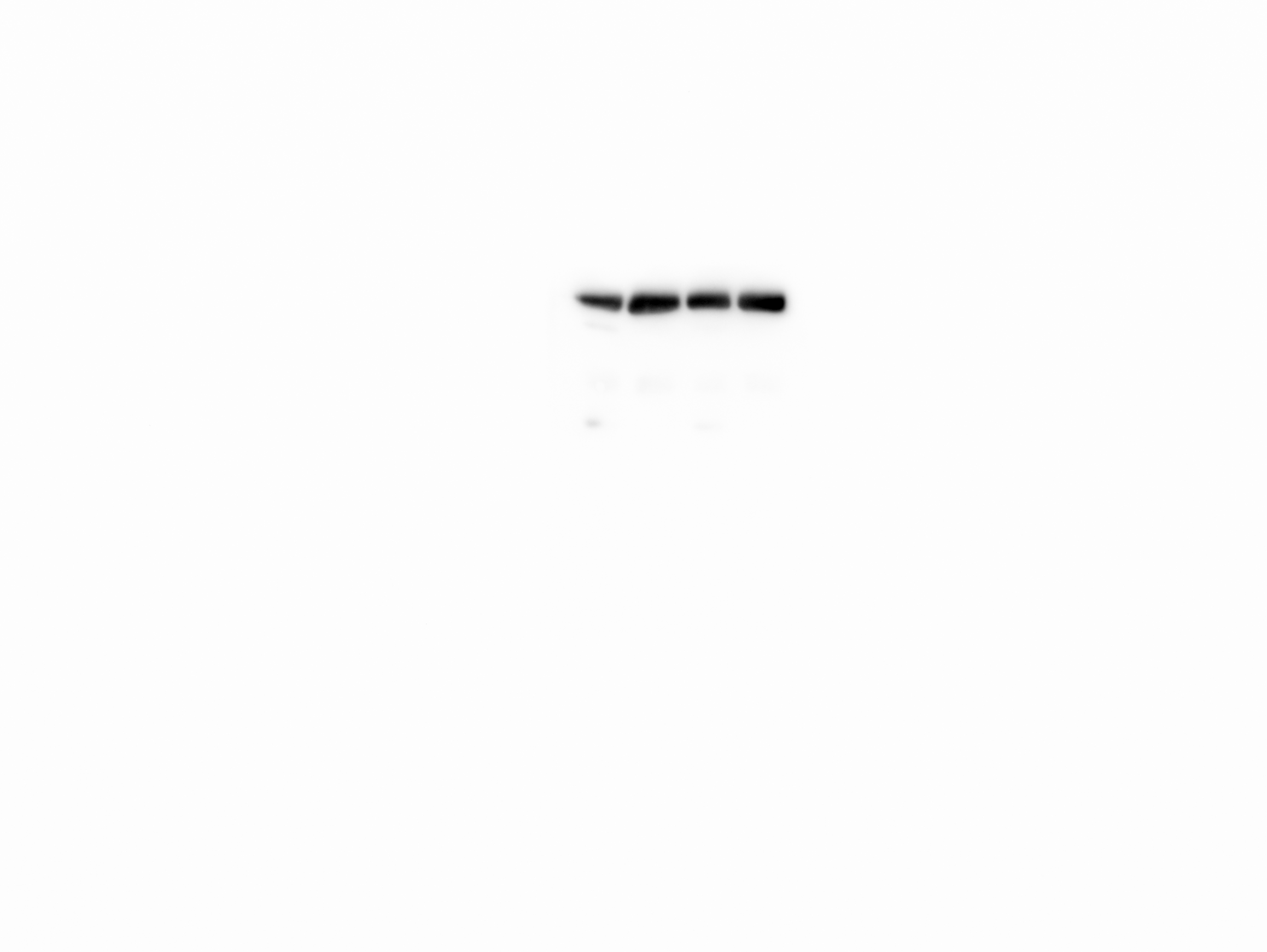

Supplement: Supplementary file 1 [file ijms-24-16899-s001.zip › WB_Whole Gels/Figure 3/a_Tubulina_U937_10%_Blot.tif]

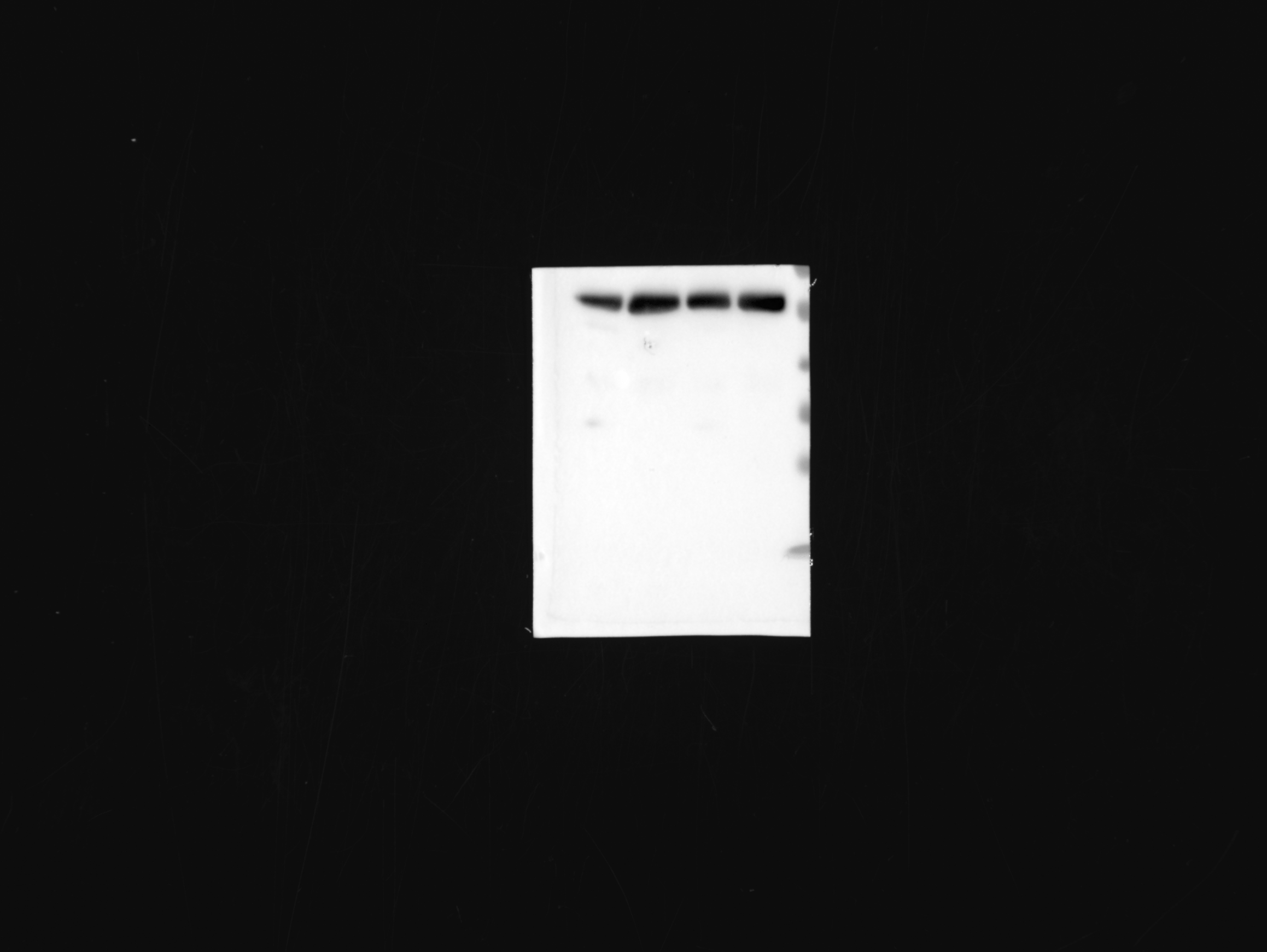

Supplement: Supplementary file 1 [file ijms-24-16899-s001.zip › WB_Whole Gels/Figure 3/a_Tubulina_U937_10%_Marker.tif]

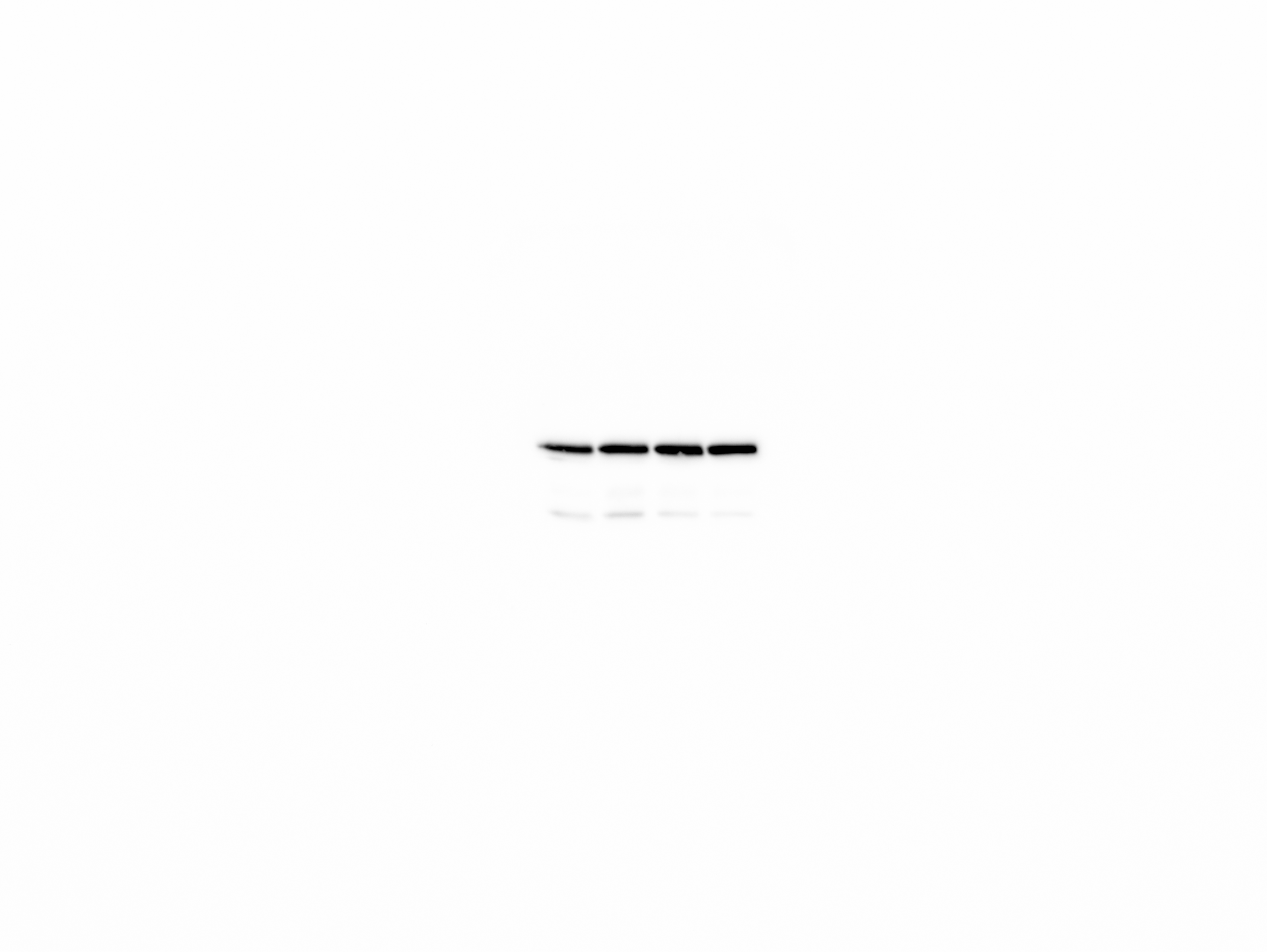

Supplement: Supplementary file 1 [file ijms-24-16899-s001.zip › WB_Whole Gels/Figure 3/a_Tubulina_U937_15%_Blot.tif]

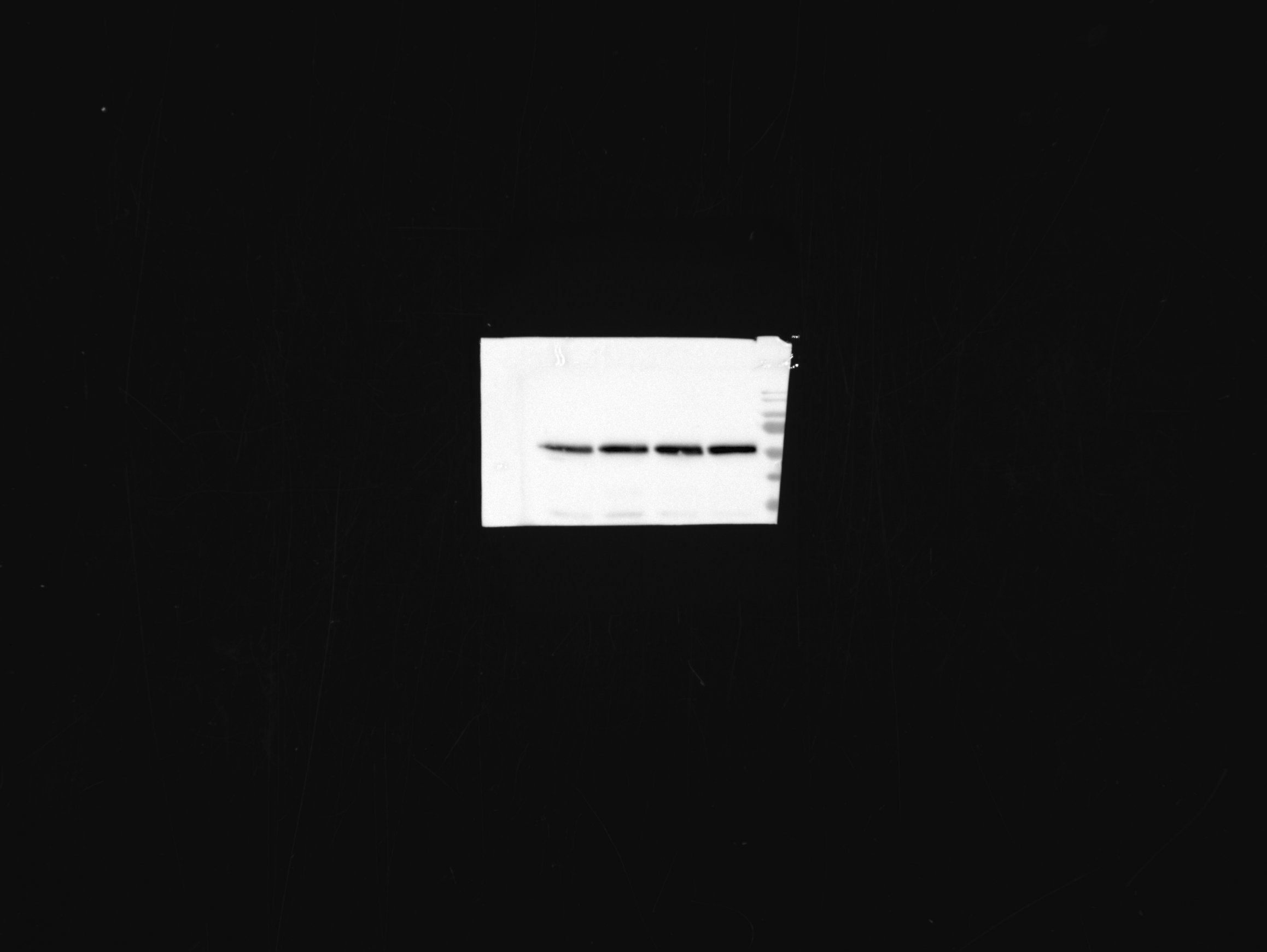

Supplement: Supplementary file 1 [file ijms-24-16899-s001.zip › WB_Whole Gels/Figure 3/a_Tubulina_U937_15%_Marker.tif]

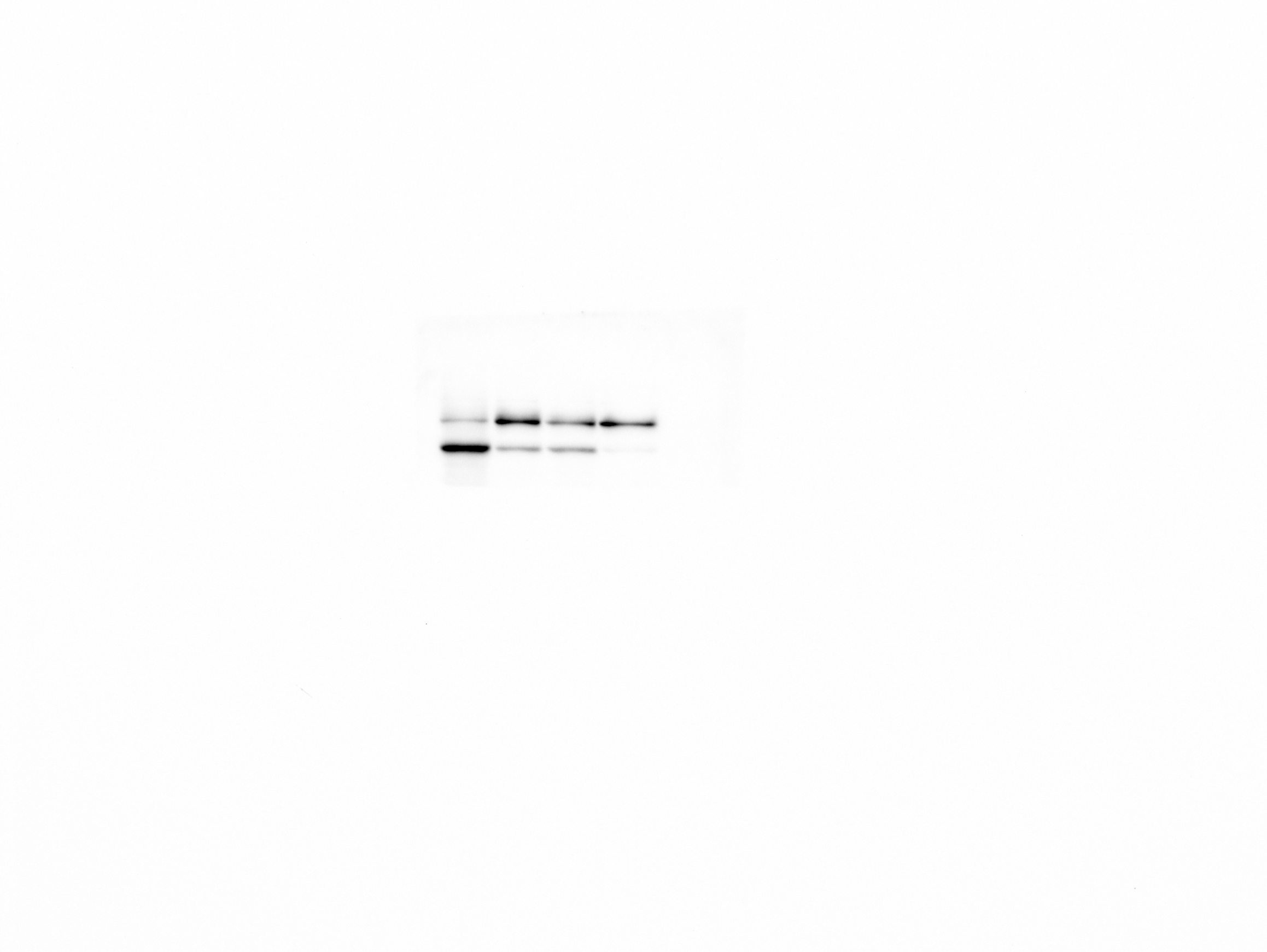

Supplement: Supplementary file 1 [file ijms-24-16899-s001.zip › WB_Whole Gels/Figure 3/PARP1_KS1_10%_Blot.tif]

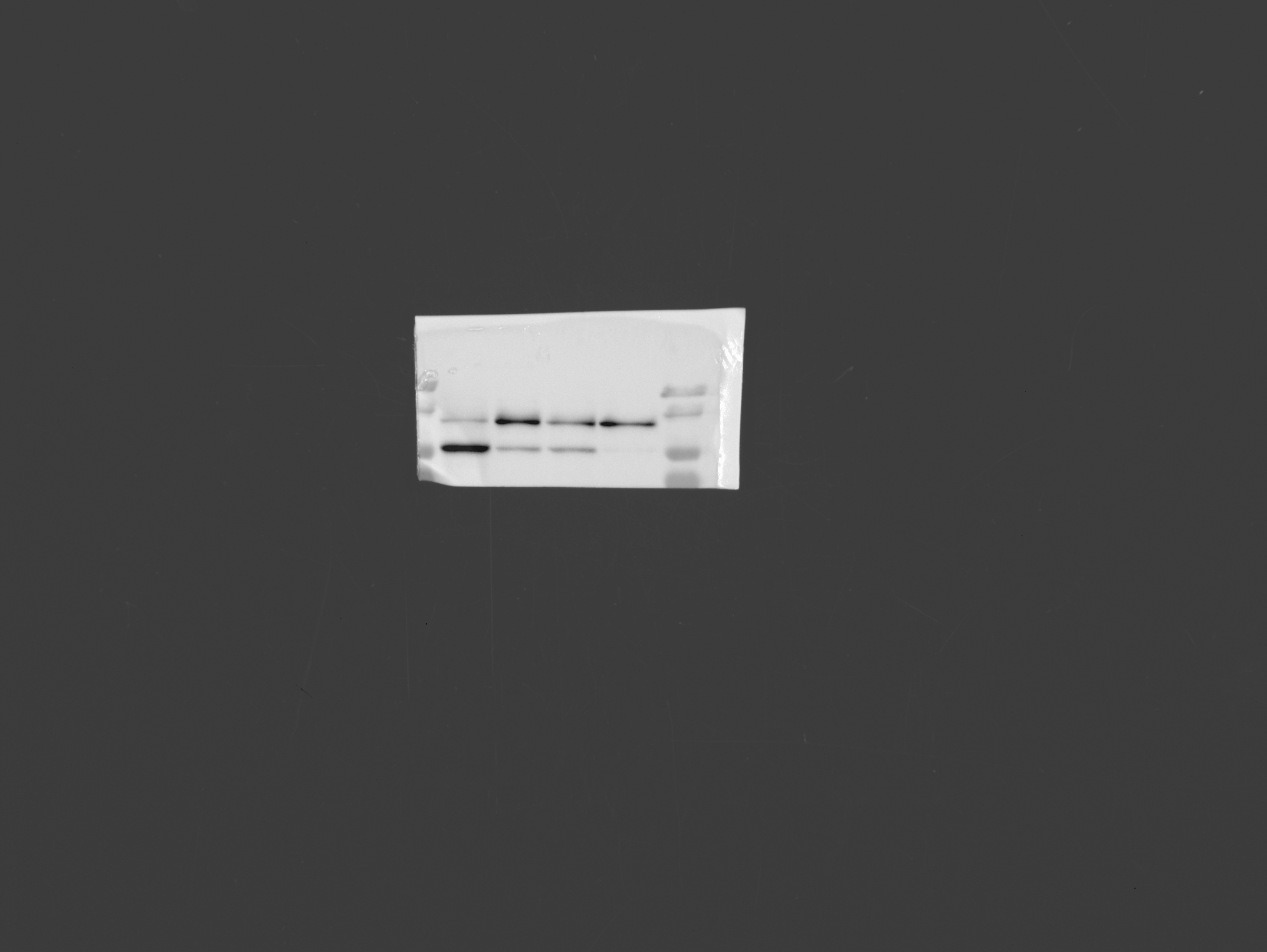

Supplement: Supplementary file 1 [file ijms-24-16899-s001.zip › WB_Whole Gels/Figure 3/PARP1_KS1_10%_Marker.tif]

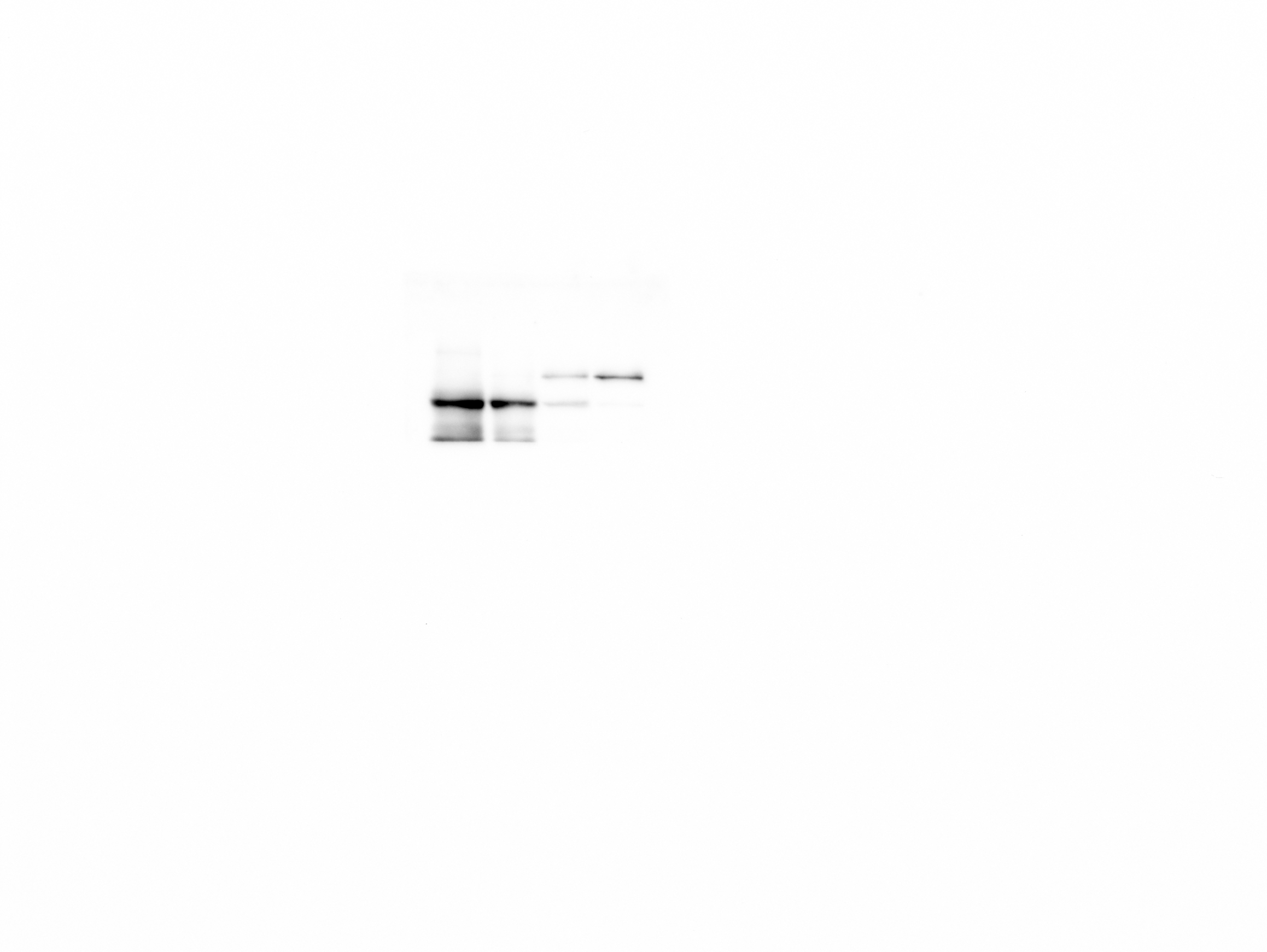

Supplement: Supplementary file 1 [file ijms-24-16899-s001.zip › WB_Whole Gels/Figure 3/PARP1_NB4_10%_Blot.tif]

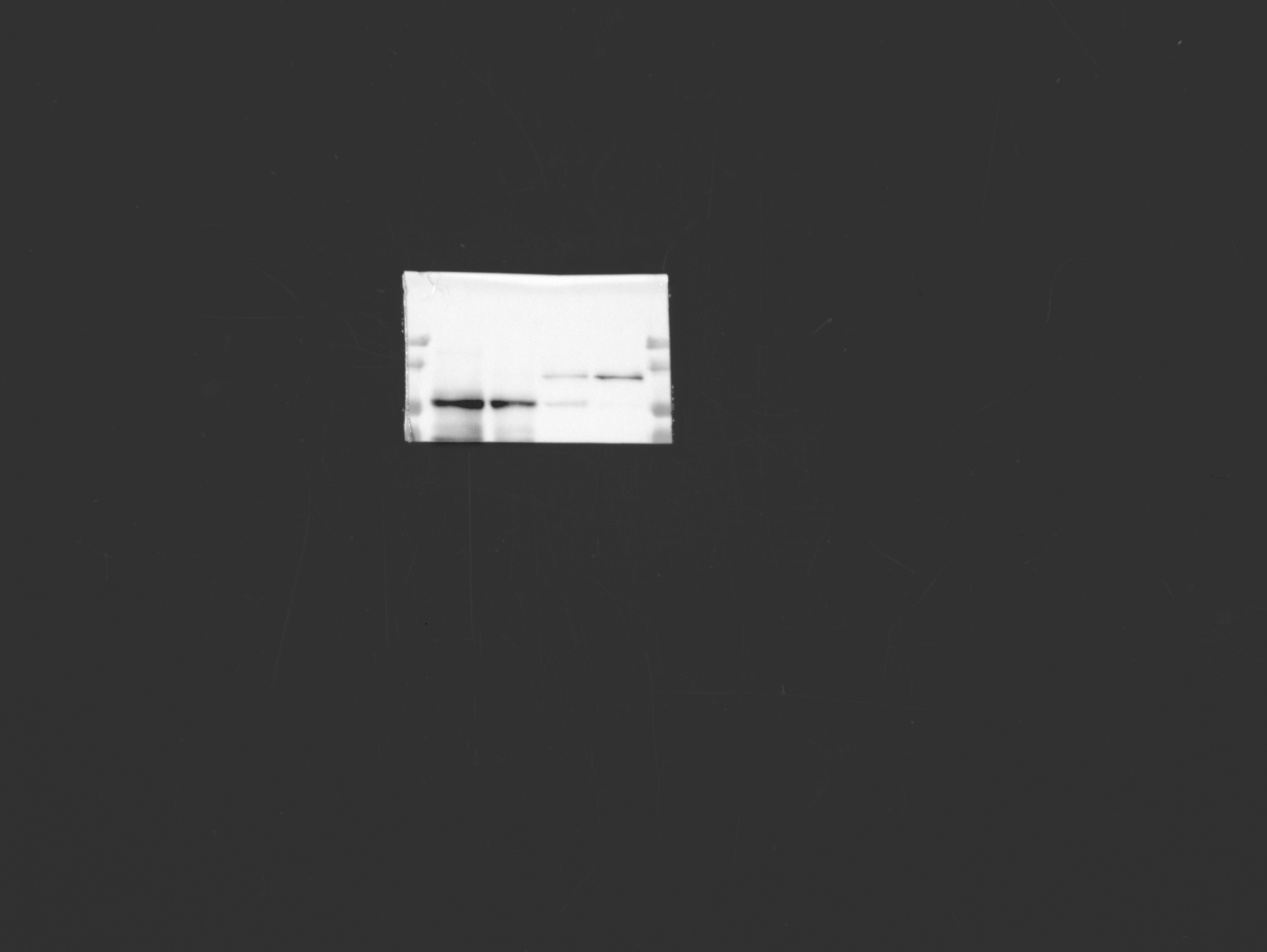

Supplement: Supplementary file 1 [file ijms-24-16899-s001.zip › WB_Whole Gels/Figure 3/PARP1_NB4_10%_Marker.tif]

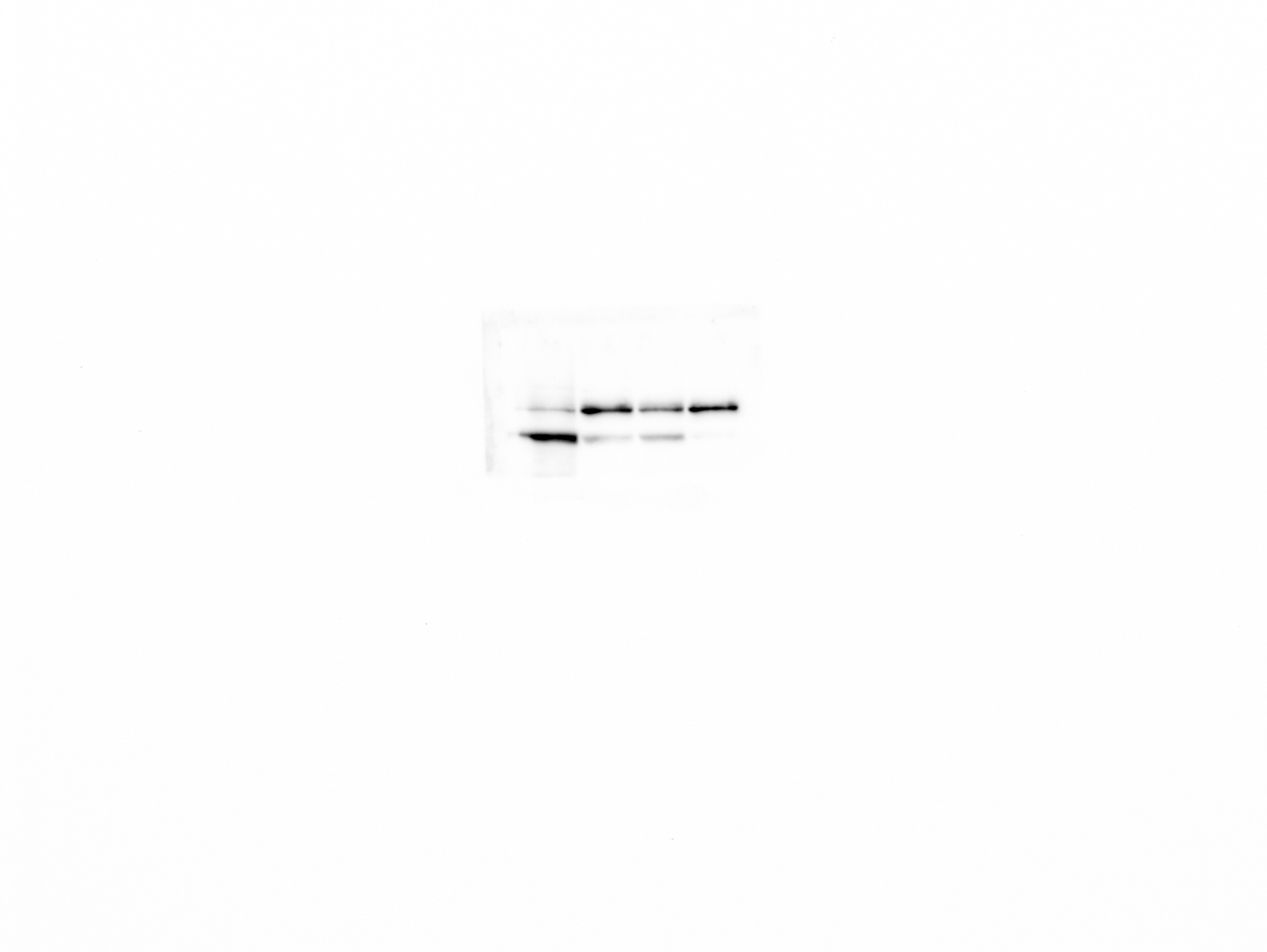

Supplement: Supplementary file 1 [file ijms-24-16899-s001.zip › WB_Whole Gels/Figure 3/PARP1_U937_10%_Blot.tif]

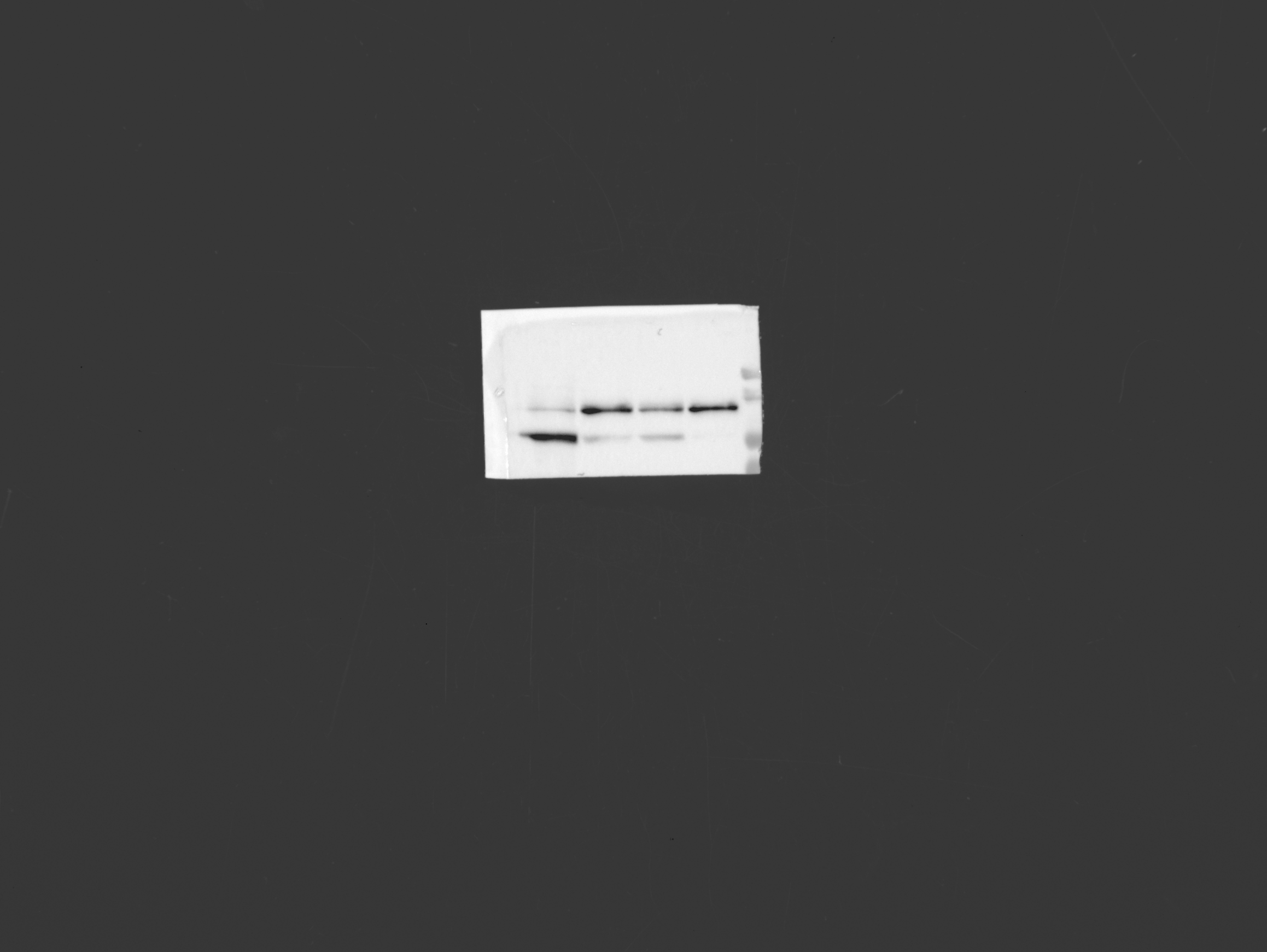

Supplement: Supplementary file 1 [file ijms-24-16899-s001.zip › WB_Whole Gels/Figure 3/PARP1_U937_10%_Marker.tif]

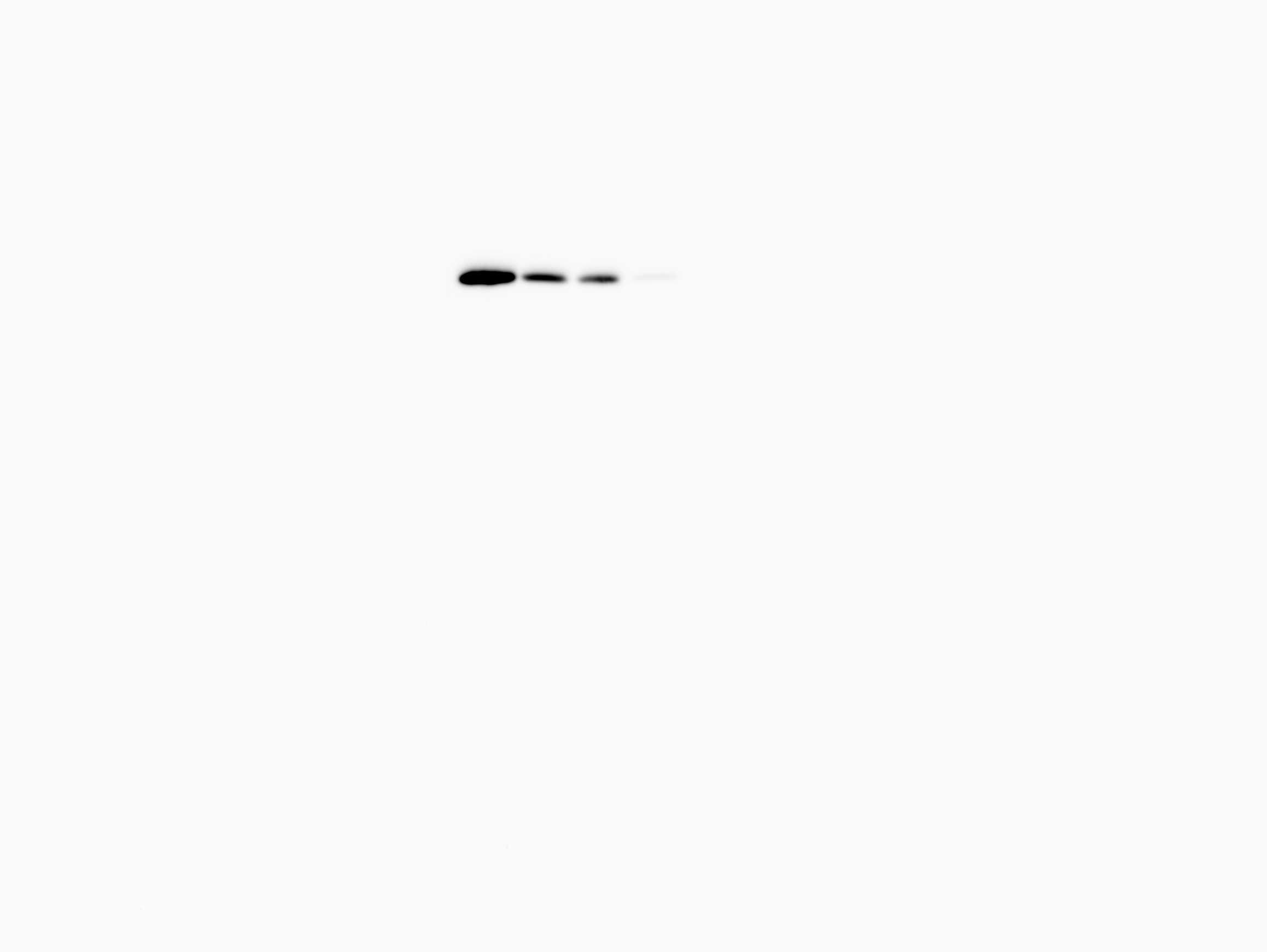

Supplement: Supplementary file 1 [file ijms-24-16899-s001.zip › WB_Whole Gels/Figure 3/YH2AX_KS1_15%_Blot.tif]

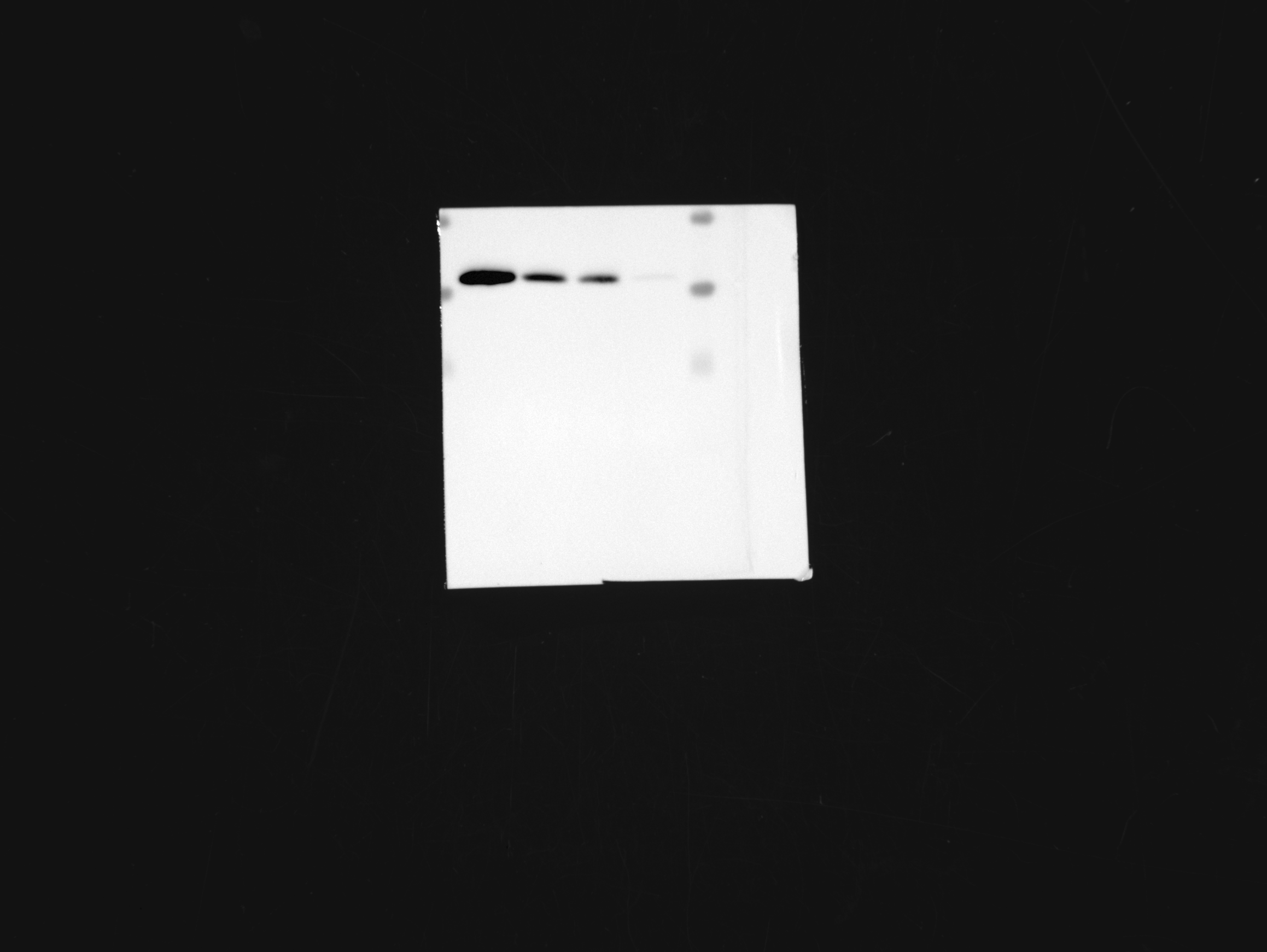

Supplement: Supplementary file 1 [file ijms-24-16899-s001.zip › WB_Whole Gels/Figure 3/YH2AX_KS1_15%_Marker.tif]

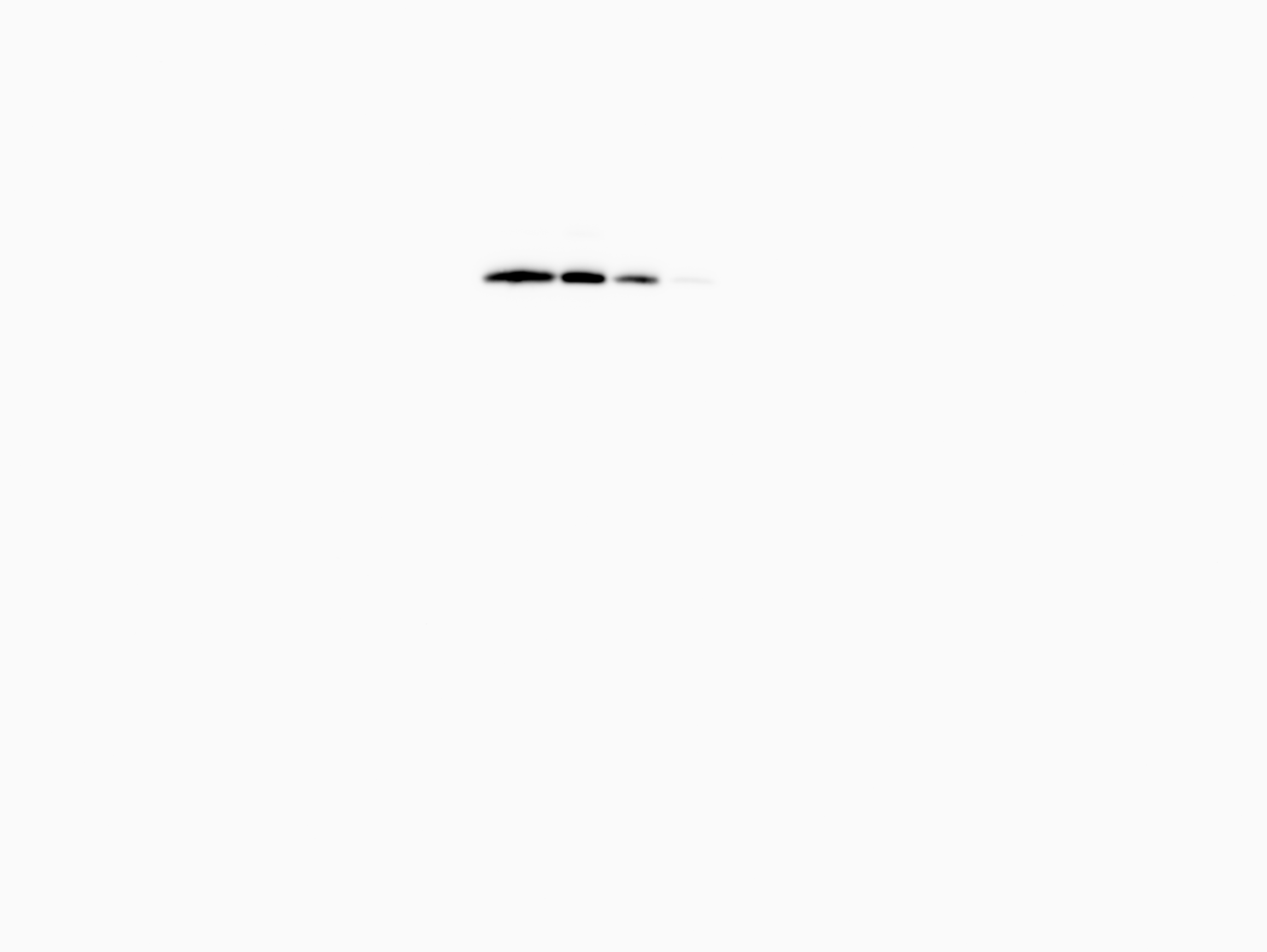

Supplement: Supplementary file 1 [file ijms-24-16899-s001.zip › WB_Whole Gels/Figure 3/YH2AX_NB4_15%_Blot.tif]

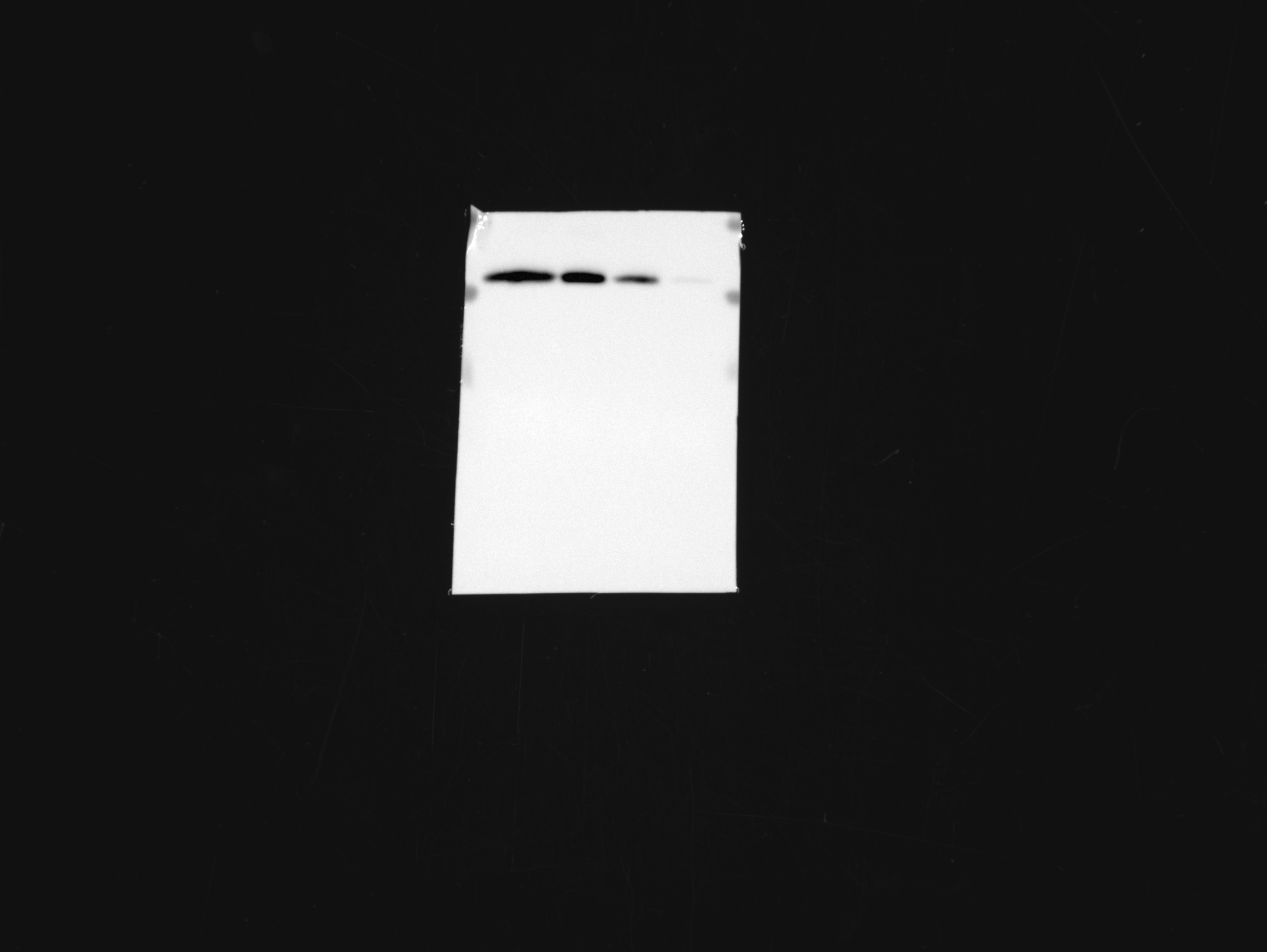

Supplement: Supplementary file 1 [file ijms-24-16899-s001.zip › WB_Whole Gels/Figure 3/YH2AX_NB4_15%_Marker.tif]

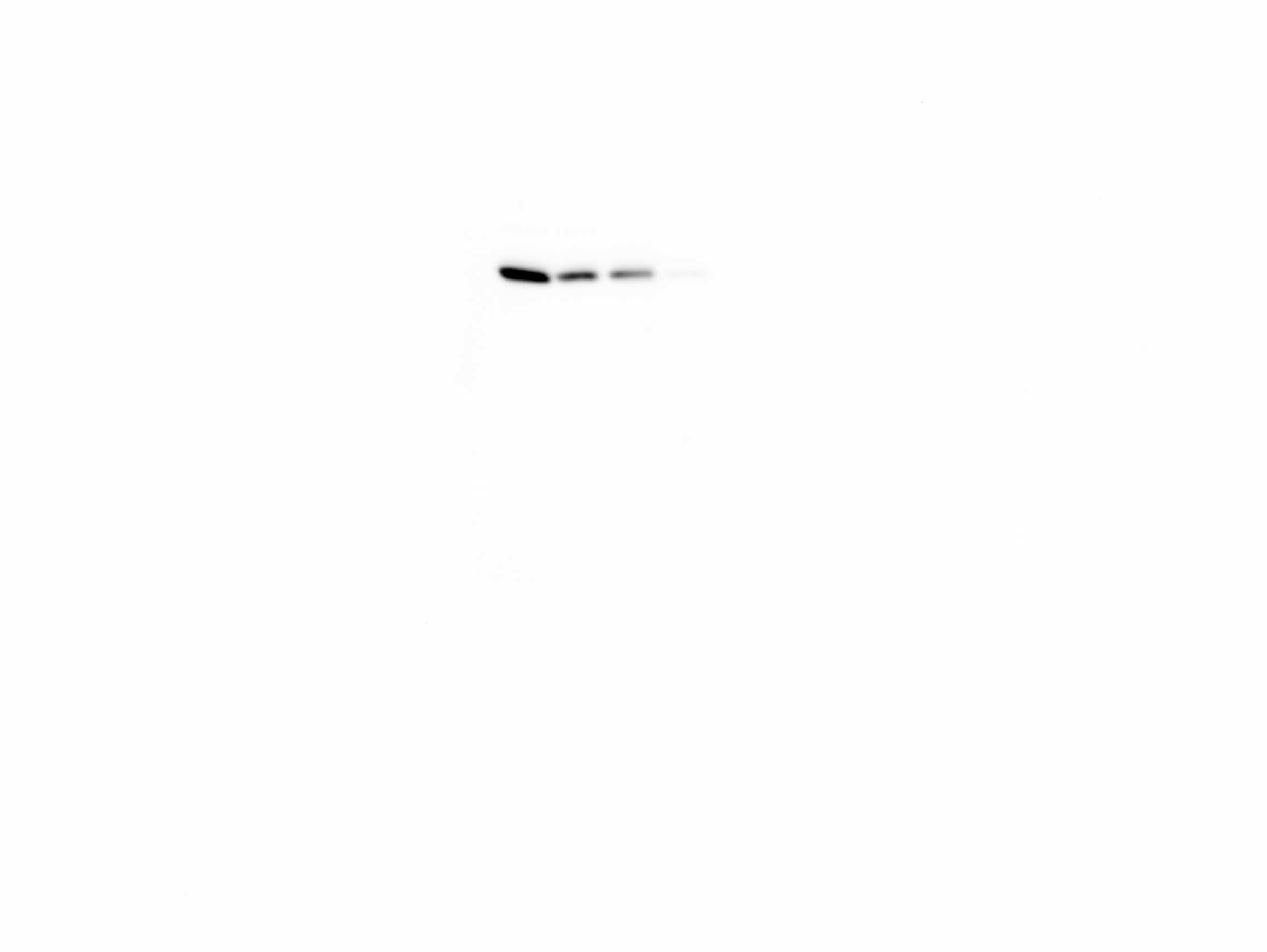

Supplement: Supplementary file 1 [file ijms-24-16899-s001.zip › WB_Whole Gels/Figure 3/YH2AX_U937_15%_Blot.sgd.tif]

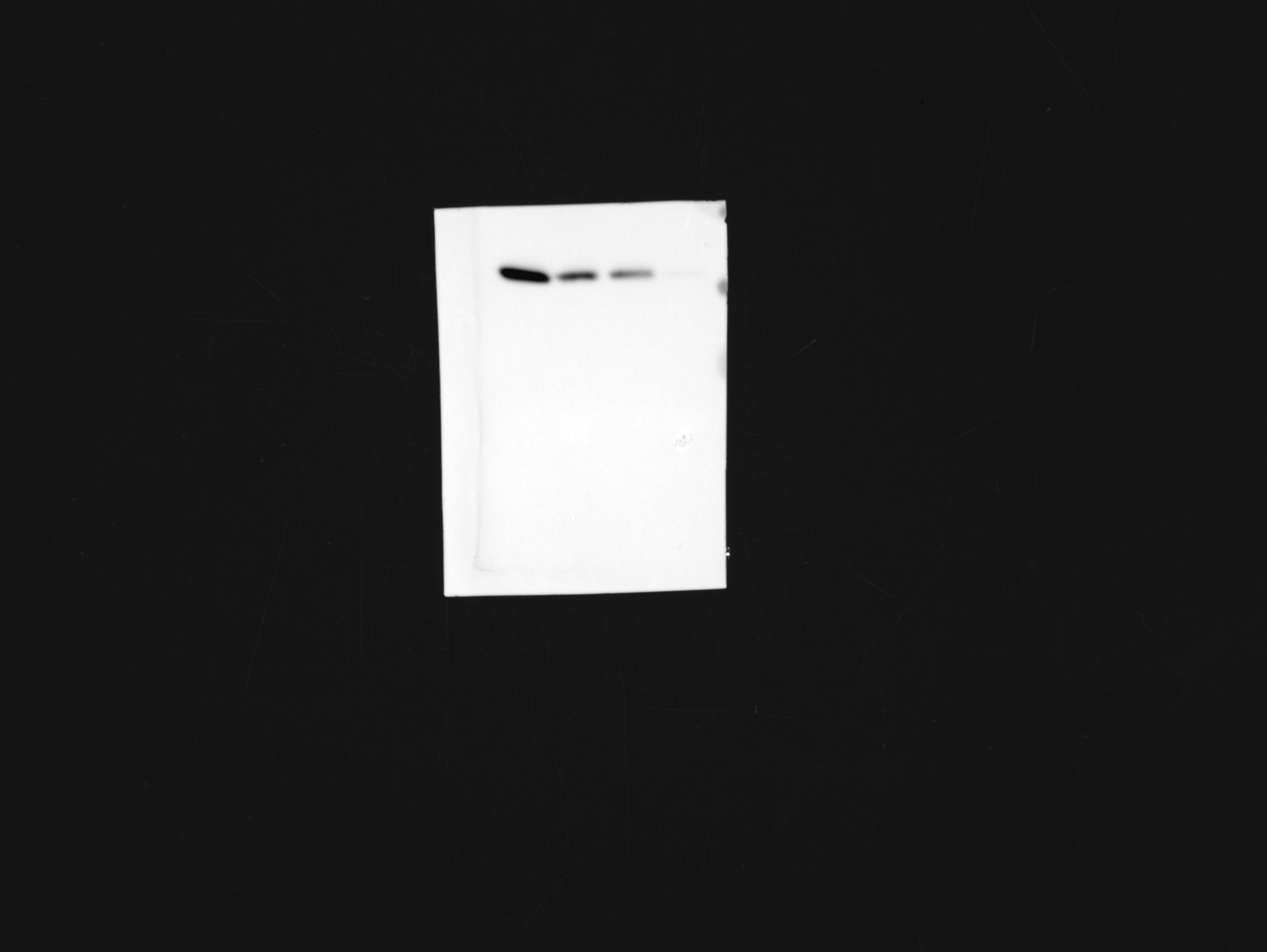

Supplement: Supplementary file 1 [file ijms-24-16899-s001.zip › WB_Whole Gels/Figure 3/YH2AX_U937_15%_Marker.tif]
